# Restoration of type 1 iodothyronine deiodinase expression in renal cancer cells downregulates oncoproteins and affects key metabolic pathways as well as anti-oxidative system

**DOI:** 10.1371/journal.pone.0190179

**Published:** 2017-12-22

**Authors:** Piotr Popławski, Jacek R. Wiśniewski, Eddy Rijntjes, Keith Richards, Beata Rybicka, Josef Köhrle, Agnieszka Piekiełko-Witkowska

**Affiliations:** 1 Department of Biochemistry and Molecular Biology, Centre of Postgraduate Medical Education, Warsaw, Poland; 2 Biochemical Proteomics Group, Max-Planck-Institute of Biochemistry, Martinsried, Germany; 3 Institut für Experimentelle Endokrinologie, Charité-Universitätsmedizin Berlin, Berlin, Germany; University Claude Bernard Lyon 1, FRANCE

## Abstract

Type 1 iodothyronine deiodinase (DIO1) contributes to deiodination of 3,5,3’,5’-tetraiodo-L-thyronine (thyroxine, T4) yielding of 3,5,3’-triiodothyronine (T3), a powerful regulator of cell differentiation, proliferation, and metabolism. Our previous work showed that loss of DIO1 enhances proliferation and migration of renal cancer cells. However, the global effects of DIO1 expression in various tissues affected by cancer remain unknown. Here, the effects of stable DIO1 re-expression were analyzed on the proteome of renal cancer cells, followed by quantitative real-time PCR validation in two renal cancer-derived cell lines. DIO1-induced changes in intracellular concentrations of thyroid hormones were quantified by L-MS/MS and correlations between expression of DIO1 and potential target genes were determined in tissue samples from renal cancer patients. Stable re-expression of DIO1, resulted in 26 downregulated proteins while 59 proteins were overexpressed in renal cancer cells. The ‘downregulated’ group consisted mainly of oncoproteins (e.g. STAT3, ANPEP, TGFBI, TGM2) that promote proliferation, migration and invasion. Furthermore, DIO1 re-expression enhanced concentrations of two subunits of thyroid hormone transporter (SLC7A5, SLC3A2), enzymes of key pathways of cellular energy metabolism (e.g. TKT, NAMPT, IDH2), sex steroid metabolism and anti-oxidative response (AKR1C2, AKR1B10). DIO1 expression resulted in elevated intracellular concentration of T4. Expression of DIO1-affected genes strongly correlated with DIO1 transcript levels in tissue samples from renal cancer patients as well as with their poor survival. This first study addressing effects of deiodinase re-expression on proteome of cancer cells demonstrates that induced DIO1 re-expression in renal cancer robustly downregulates oncoproteins, affects key metabolic pathways, and triggers proteins involved in anti-oxidative protection. This data supports the notion that suppressed DIO1 expression and changes in local availability of thyroid hormones might favor a shift from a differentiated to a more proliferation-prone state of cancer tissues and cell lines.

## Introduction

Clear cell renal cell carcinoma (ccRCC) is the most common subtype of kidney tumors, affecting more than 300,000 people annually worldwide [1]. The key molecular alteration in ccRCC pathology is inactivation of VHL tumor suppressor that leads to persistent activation of hypoxia-induced transcription factors (HIFs), resulting in induction of proliferation, invasion and angiogenesis [2]. Recent findings indicate that tumorous phenotype of ccRCC is largely driven by alterations in cellular metabolism [3–5]. They include Warburg effect, the universal feature of cancer cells, that is defined as increased consumption of glucose which is largely converted to lactate, even under normoxic conditions, as well as activated pentose phosphate pathway (PPP), suppressed TCA cycle, enhanced lipogenesis and metabolism of amino acids [5–8]. These changes are interpreted as a metabolic reprogramming that enables efficient production of essential building blocks (nucleotides, lipids, amino acids) required to sustain intensive proliferation of cancer cells. This metabolic shift also provides large amounts of metabolites which contribute to cellular buffering system and protection against acidic environment and oxidative stress of tumors [6].

Type 1 iodothyronine deiodinase (DIO1) is one of the three enzymes regulating bioavailability of thyroid hormones in thyroid and extrathyroid tissues [9,10]. By catalyzing deiodination of thyroxine (T4), DIO1 can contribute to the synthesis of 3,5,3’triiodo-L-thyronine (T3), a powerful regulator of cellular differentiation, proliferation, metabolism, and apoptosis acting via classical T3 receptors and non-classical rapid signaling [11–13]. The expression of deiodinase isoenzymes (DIO1, DIO2, DIO3) is altered in cancers, providing dynamic changes in intracellular steady state T3 concentrations which impact on the expression of T3-dependent genes and contribute to processes involved in cancerogenesis. Previous studies on DIO2 and DIO3 in colon cancer and basal cell carcinoma revealed that their altered enzyme activities caused changes in expression of genes involved in tumor development, progression and apoptosis [14–18]. Our recent work revealed that reduced expression of DIO1 in renal cancer contributes to altered expression of genes controlling cell cycle progression, adhesion and migration, with marked impact on proliferation and cell motility [19–21]. Remarkably, although the regulation of DIO1 expression is relatively well understood [11,22–24], the global effects of DIO1 expression in cancer remain unknown. Considering the importance of T3 in the regulation of cellular metabolism, one can also expect, that changes in DIO1 expression could affect metabolic pathways in ccRCC tumors.

To get more mechanistic insight into the effects of DIO1 expression in cancer cells, the current study reports on proteomic analysis of renal cancer cells which were stably transfected by a vector expressing DIO1. The presence of DIO1 activity in renal cancer cells introduces major changes in cellular proteome, affecting i) the key metabolic pathways that are altered in ccRCC tissues, ii) the elements of the anti-oxidative system as well as iii) expression levels of proteins that drive oncogenic transformation of renal cells.

## Materials and methods

### Human cell lines

KIJ265T and KIJ308T cell lines, derived from ccRCC, were obtained from Mayo Foundation for Medical Education and Research [25] and cultured as described previously [26]. Preparation of cells stably transfected with pcDNA3-DIO1 plasmid (kindly provided by T.J. Visser, [27]) or with an empty vector, was described previously [21]. Expression of DIO1 was determined by qPCR and Western blot.

For intracellular hormone measurements, KIJ265T-DIO1(+) and KIJ265-DIO1(-) cells were cultured in medium without phenol red supplemented with 10% FBS (Sigma-Aldrich, St. Louis, MO, USA) for 7 days. The mean T4 and T3 concentrations in FBS-supplemented cell culture medium was 26.2 nM and 0.069 nM, respectively, which is within the range of concentrations reported previously [28–30]. Next, cells were seeded on 6-well plates at density 1.25×10^5^ per well and the medium was renewed after 24h. The cells were cultured for the next 24h with medium renewal after 24h. Following the next 24h, the medium was collected, and the plates with adhered cells were put on ice, washed with ice-cold PBS and stored in -80°C until analysis.

### Thyroid hormone analysis

The sample preparation and analysis of supernatants of cell culture medium [31] and cells [32] has been previously described. In brief, cell culture plate wells containing dry cells were treated with lysis buffer (0.1 N sodium hydroxide and homogenization buffer 50:50% v/v, 200 μL) on ice for 3 min with shaking; 30% v/v acetic acid in homogenization buffer (100 μL) was then added. Cell culture medium supernatants (400 μL) were acidified with 37% HCl (5 μL). Both cell lysates and cell culture medium supernatants were spiked with a mixture of internal standards (for supernatants: in 5 μL DMSO, for cell lysates: in 100 μL in DMSO: methanol: water 5:45:45% v/v/v containing 0.1% formic acid) composed of isotopically-labelled 3,3-T_2_, T_3_, rT_3_,T_4_, 3-T_1_AM, T_0_Ac and T_1_Ac at a final concentration of 100 nM. Internal standard-spiked supernatant or cell lysates were then incubated at 37°C for 60 min, then twice liquid-liquid extracted (2 x 1 mL of 30% v/v 2-propanol in t-butyl methyl ether, combining the 2 x 1mL extracts). The extracts were evaporated to dryness, then re-constituted in 50:50% v/v methanol: water containing 0.1% formic acid (100 μL). 40 μL of extract was injected into a Sciex API 6500 QTRAP LC-MS/MS system equipped with a 100 x 3 mm Waters X-Select HSS PFP column, running a water (UHP, 18.2 mΩ) /methanol (containing 0.1% formic acid) gradient. The mass spectrometer was operated in electrospray positive ionization mode using multiple reaction monitoring (MRM). Data was acquired and processed with Analyst™ 1.6.2 and MultiQuant™ 2.11 software. Linear calibration curves in cell culture medium were obtained in the range from 0.0125 to 250 nM.

### Proteomic analysis

For protein isolations, the cells were seeded on 6-well plates at density of 1.25 × 10^5^ cells per well and cultured for 72 hours. Medium was removed and cells were rinsed 3 times with PBS without calcium and magnesium (Thermo Fisher Scientific, Rockford, IL, USA). Next, cells were lysed with 500 μl of protein isolation buffer (0.1M Tris pH 7.5, 2% SDS, 0.1M DTT) and boiled for 5 min. Protein lysates were processed using the MED/FASP procedure and the resulting peptide digests were analyzed as described previously [33,34]. Total protein in the SDS lysates and peptide concentration in the digests were determined using the WF assay [35]. Titers of protein were calculated by the 'total protein approach' using the raw spectral intensities from MaxQuant output [36].

Protein enrichment analysis and classification according to molecular functions, cellular localizations and classes was performed using http://www.geneontology.org/ [37] powered by Protein Analysis Through Evolutionary Relationships (PANTHER) [38]. Protein-protein interaction network was generated using STRING v. 10.5 with default settings (minimum required interaction score: medium confidence 0.4) [39].

### Isolation of RNA and cDNA synthesis

For RNA isolations, the cells were seeded on 12-well plates at density of 5 × 10^4^ cells per well and cultured for 72 hours. RNA was isolated using GeneMATRIX Universal RNA Purification Kit (EURx, Gdańsk, Poland) and reverse transcription was performed as described previously [21].

RNA samples from human ccRCC and matched-paired control tissues not infiltrated by tumor were obtained from the RNA Bank deposited at the Centre of Postgraduate Medical Education at the Department of Biochemistry and Molecular Biology (approved by the local Bioethical Committee: no. 18/PB/2012 and no. 75/PB-A/2014). 1000 ng of RNA was reverse transcribed as described previously [21] with Transcriptor First Strand cDNA Synthesis Kit (Roche Diagnostics, Mannheim, Germany) using random hexamer primers and anchored-oligo(dT)_18_.

### Real-time quantitative PCR

Real-time quantitative PCR (qPCR) was performed on LightCycler® 480 (Roche Diagnostics) using primers and probes given in S1 Table and SYBR Green I Master (Roche Diagnostics) or TaqManUniversal Master MiX II with UNG (Thermo Fisher Scientific), according to producers protocols. Reference genes for normalization were chosen using Normfinder tool [40].

### Protein isolation and Western blotting

Protein isolations were performed as described previously [21, 41]. 60 μg of proteins was resolved using 10% SDS-PAGE and Western blot analysis was performed as described previously [21].

### Survival analysis

Survival rate analysis was performed as described previously [41] using the SurvExpress platform [42] on transcriptomic data of independent cohort of ccRCC patients, retrieved from TCGA (Cancer Genome Atlas Network, https://tcga-data.nci.nih.gov, [43]). The median follow up of the 468 TCGA ccRCC patients was 43.2 months. The relationship between the gene expression and survival time was estimated using Cox Proportional Hazard regression. Two risk groups were generated using the prognostic index median. The equality of survival curves was evaluated using log-rank test.

### Statistical analysis

Statistical analysis was performed with GraphPad Prism 5.00 for Windows (GraphPad Software, San Diego, CA, USA) using the Shapiro-Wilk normality test, Wilcoxon matched pair signed rank test, paired *t*-test and Spearman rank correlation test. *p*<0.05 was considered statistically significant.

In proteomic analysis non-paired Student's t-test was used for comparisons between two experimental groups. The maximal number of 0-values per group was 2. The missing values were imputed using the parameter values of 0.3 for width and 1.8 for down shift. *p*<0.05 was considered statistically significant.

## Results

### Identification and quantitative analysis of proteins affected by DIO1 expression

Induction of DIO1 expression was confirmed using Western blot (S1 Fig). Proteomic analysis of KIJ265T-DIO1(+) and KIJ265T-DIO1(-) cells identified peptides derived from 4761 proteins (S2 Table). Differential analysis revealed 85 proteins that were differently expressed in KIJ265T-DIO1(+) cells when compared with KIJ265T-DIO1(-) cells (False Discovery Rate<0.05, p<0.05). Among these proteins, 26 were upregulated (Table 1), while 59 were downregulated (Table 2) in DIO1(+) cells. Top upregulated proteins included AKR1C2 (+57 fold), RAP1GAP (+15.8 fold), and AKR1B10 (+8.2 fold). Top proteins with decreased expression included AFAP1L2 (-31.2 fold), ANPEP (-8.7 fold), and CYR61 (-8.2 fold). Remarkably, among the proteins with expression increased there were two subunits of LAT1 transporter (SLC7A5 (+3.66 fold) and SLC3A2 (+1.73 fold)) that is involved in intracellular transport of amino acids and thyroid hormones [44].

**Table 1:** The proteins upregulated in human renal cancer cell with induced DIO1 expression.

Gene name	Protein name	Fold change (DIO1+/DIO1- ratio)	P value
*AKR1C2*	Aldo-keto reductase family 1 member C2	57.01	4.18×10^-4^
*RAP1GAP*	Rap1 GTPase-activating protein 1	15.84	1.52×10^-5^
*AKR1B10*	Aldo-keto reductase family 1 member B10	8.20	5.40×10^-8^
*PPIF*	Peptidyl-prolyl cis-trans isomerase F, mitochondrial	3.86	5.65×10^-5^
*ABCB6*	ATP-binding cassette sub-family B member 6, mitochondrial	3.82	1.35×10^-3^
*AKR1C1*	Aldo-keto reductase family 1 member C1	3.78	7.73×10^-5^
*SLC7A5*	Large neutral amino acids transporter small subunit 1	3.66	4.80×10^-4^
*TAGLN*	Transgelin	3.34	5.86×10^-7^
*AKR1C3*	Aldo-keto reductase family 1 member C3	2.77	3.54×10^-4^
*CYP4F11*	Phylloquinone omega-hydroxylase CYP4F11	2.52	1.42×10^-5^
*UBAC2*	Ubiquitin-associated domain-containing protein 2	2.43	9.59×10^-4^
*FSTL1*	Follistatin-related protein 1	2.40	4.51×10^-5^
*MFGE8*	Lactadherin; Lactadherin short form; Medin	2.31	5.21×10^-4^
*FAH*	Fumarylacetoacetase	2.25	5.93×10^-4^
*UGT1A6*	UDP-glucuronosyltransferase 1-6	1.99	4.44×10^-4^
*GCLC*	Glutamate-cysteine ligase catalytic subunit	1.97	8.34×10^-4^
*UGDH*	UDP-glucose 6-dehydrogenase	1.94	3.33×10^-4^
*PSMB5*	Proteasome subunit beta type-5	1.90	1.17×10^-3^
*EIF4A2*	Eukaryotic initiation factor 4A-II; Eukaryotic initiation factor 4A-II, N-terminally processed	1.76	6.33×10^-4^
*SLC3A2*	4F2 cell-surface antigen heavy chain	1.73	9.44×10^-5^
*NAMPT*	Nicotinamide phosphoribosyltransferase	1.57	2.89×10^-5^
*TKT*	Transketolase	1.57	5.21×10^-4^
*PLOD2*	Procollagen-lysine,2-oxoglutarate 5-dioxygenase 2	1.56	6.13×10^-4^
*IDH2*	Isocitrate dehydrogenase [NADP], mitochondrial	1.52	1.21×10^-3^
*TMX2*	Thioredoxin-related transmembrane protein 2	1.40	7.05×10^-4^
*CRYZ*	Quinone oxidoreductase	1.33	1.62×10^-4^

The table shows results of proteomic analysis performed in KIJ265T cells transfected with pcDNA3-DIO1 or empty plasmid. Only proteins of which levels were statistically significantly increased in DIO1 expressing cells (DIO1+) when compared with cells transfected with an empty plasmid (DIO1-) are shown (threshold: 1.3-fold change, FDR<0.05, p<0.05). The raw proteomic data are given in S2 Table.

**Table 2:** The proteins downregulated in human renal cancer cell with induced DIO1 expression.

Gene name	Protein name	Fold change (DIO1+/DIO1-) ratio)	P value
*AFAP1L2*	Actin filament-associated protein 1-like 2	-31.22	1.73×10^-5^
*ANPEP*	Aminopeptidase N	-8.74	3.47×10^-6^
*CYR61*	Protein CYR61	-8.25	2.47×10^-7^
*NMT2*	Glycylpeptide N-tetradecanoyltransferase 2; Glycylpeptide N-tetradecanoyltransferase	-6.80	6.43×10^-4^
*MICAL3*	Protein-methionine sulfoxide oxidase MICAL3	-5.04	1.72×10^-5^
*PLAU*	Urokinase-type plasminogen activator; Urokinase-type plasminogen activator long chain A; Urokinase type plasminogen activator short chain A; Urokinase-type plasminogen activator chain B	-4.59	1.08×10^-3^
*WIZ*	Protein Wiz	-4.24	6.16×10^-4^
*ANXA3*	Annexin A3; Annexin	-3.90	1.66×10^-4^
*PLCB4*	1-phosphatidylinositol 4,5-bisphosphate phosphodiesterase beta-4	-3.83	1.08×10^-5^
*FMNL2*	Formin-like protein 2	-3.57	6.15×10^-5^
*FHL1*	Four and a half LIM domains protein 1	-3.49	5.66×10^-5^
*APBB1IP*	Amyloid beta A4 precursor protein-binding family B member 1-interacting protein	-3.40	6.03×10^-5^
*ASAP1*	Arf-GAP with SH3 domain, ANK repeat and PH domain-containing protein 1	-3.29	1.00×10^-3^
*LRRFIP1*	Leucine-rich repeat flightless-interacting protein 1	-3.28	8.30×10^-4^
*SCRN1*	Secernin-1	-3.26	8.91×10^-4^
*TGM2*	Protein-glutamine gamma-glutamyltransferase	-3.15	1.22×10^-3^
*LACTB*	Serine beta-lactamase-like protein LACTB, mitochondrial	-3.11	8.35×10^-5^
*TGFBI*	Transforming growth factor-beta-induced protein ig-h3	-3.07	6.68×10^-4^
*MAP4K5*	Mitogen-activated protein kinase kinase kinase kinase 5; Mitogen-activated protein kinase kinase kinase kinase	-2.97	1.10×10^-4^
*PODXL*	Podocalyxin	-2.73	1.48×10^-4^
*NMES1;C15orf48*	Normal mucosa of esophagus-specific gene 1 protein	-2.72	3.54×10^-4^
*CD74*	HLA class II histocompatibility antigen gamma chain	-2.63	1.10×10^-3^
*SUN2*	SUN domain-containing protein 2	-2.46	2.55×10^-4^
*RBKS*	Ribokinase	-2.39	1.27×10^-4^
*LEPREL1*	Prolyl 3-hydroxylase 2	-2.35	1.72×10^-4^
*ADAMTS1*	A disintegrin and metalloproteinase with thrombospondin motifs 1	-2.34	7.37×10^-4^
*OCIAD2*	OCIA domain-containing protein 2	-2.32	1.46×10^-4^
*DHFR*	Dihydrofolate reductase	-2.20	3.35×10^-4^
*TBC1D2*	TBC1 domain family member 2A	-2.20	2.77×10^-4^
*UBA6*	Ubiquitin-like modifier-activating enzyme 6	-2.19	3.58×10^-4^
*NMI*	N-myc-interactor	-2.10	5.18×10^-4^
*EEA1*	Early endosome antigen 1	-2.10	7.17×10^-4^
*S100A2*	Protein S100-A2	-2.03	6.19×10^-4^
*ITGAV*	Integrin alpha-V; Integrin alpha-V heavy chain; Integrin alpha-V light chain	-1.98	5.23×10^-5^
*ERAP1*	Endoplasmic reticulum aminopeptidase 1	-1.95	1.22×10^-4^
*HMGCS1*	Hydroxymethylglutaryl-CoA synthase, cytoplasmic	-1.95	3.27×10^-4^
*NDUFA3*	NADH dehydrogenase [ubiquinone] 1 alpha subcomplex subunit 3	-1.92	2.47×10^-4^
*MVP*	Major vault protein	-1.89	6.14×10^-6^
*NANS*	Sialic acid synthase	-1.88	3.58×10^-4^
*PARP4*	Poly [ADP-ribose] polymerase 4	-1.86	1.82×10^-4^
*LASP1*	LIM and SH3 domain protein 1	-1.85	3.27×10^-4^
*EPHA2*	Ephrin type-A receptor 2	-1.83	1.15×10^-3^
*ATP2C1*	Calcium-transporting ATPase type 2C member 1; Calcium-transporting ATPase	-1.83	5.26×10^-4^
*DPP9*	Dipeptidyl peptidase 9	-1.81	4.50×10^-4^
*IMMT*	MICOS complex subunit MIC60	-1.73	1.72×10^-4^
*NF2*	Merlin	-1.70	4.17×10^-4^
*STXBP2*	Syntaxin-binding protein 2	-1.66	9.78×10^-5^
*NCEH1*	Neutral cholesterol ester hydrolase 1	-1.64	8.42×10^-4^
*S100A11*	Protein S100-A11;Protein S100-A11, N-terminally processed	-1.61	1.19×10^-3^
*ENAH*	Protein enabled homolog	-1.61	3.05×10^-4^
*YWHAH*	14-3-3 protein eta	-1.60	5.87×10^-4^
*RIPK1*	Receptor-interacting serine/threonine-protein kinase 1	-1.59	1.51×10^-4^
*GLUD1;GLUD2*	Glutamate dehydrogenase 1, mitochondrial; Glutamate dehydrogenase 2, mitochondrial	-1.56	1.40×10^-3^
*AP2B1*	AP-2 complex subunit beta	-1.55	1.80×10^-4^
*DFNA5*	Non-syndromic hearing impairment protein 5	-1.53	1.09×10^-3^
*STAT3*	Signal transducer and activator of transcription 3; Signal transducer and activator of transcription	-1.52	2.30×10^-4^
*PLS3*	Plastin-3	-1.51	7.19×10^-6^
*AKR1B1*	Aldose reductase	-1.42	3.84×10^-5^
*AP3B1*	AP-3 complex subunit beta-1	-1.31	1.74×10^-4^

The table shows results of proteomic analysis performed in KIJ265T cells transfected with pcDNA3-DIO1 or empty plasmid. Only proteins of which levels were statistically significantly decreased in DIO1 expressing cells (DIO1+) when compared with cells transfected with an empty plasmid (DIO1-) are shown (threshold: 1.3-fold change, FDR<0.05, p<0.05). The raw proteomic data are given in S2 Table.

### Classification of proteins affected by DIO1 expression

**Fig 1:**
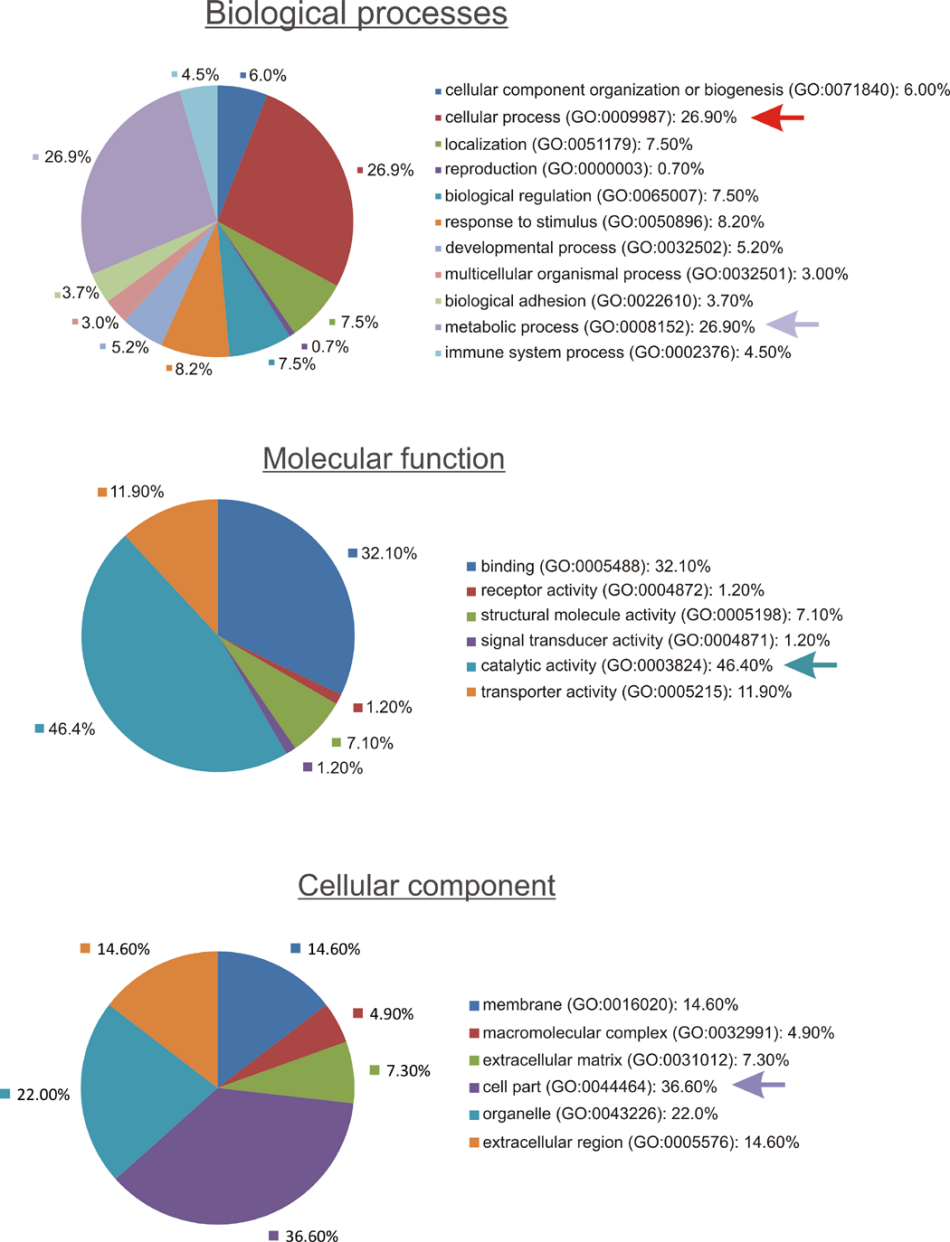
Functional annotation of proteins that were differently expressed in DIO1(+) cells when compared with DIO1(-) cells. The pie charts show results of analysis performed using PANTHER (http://pantherdb.org). The largest categories of biological processes, molecular function, and cellular components related to the identified proteins are shown with arrows.

**Fig 2:**
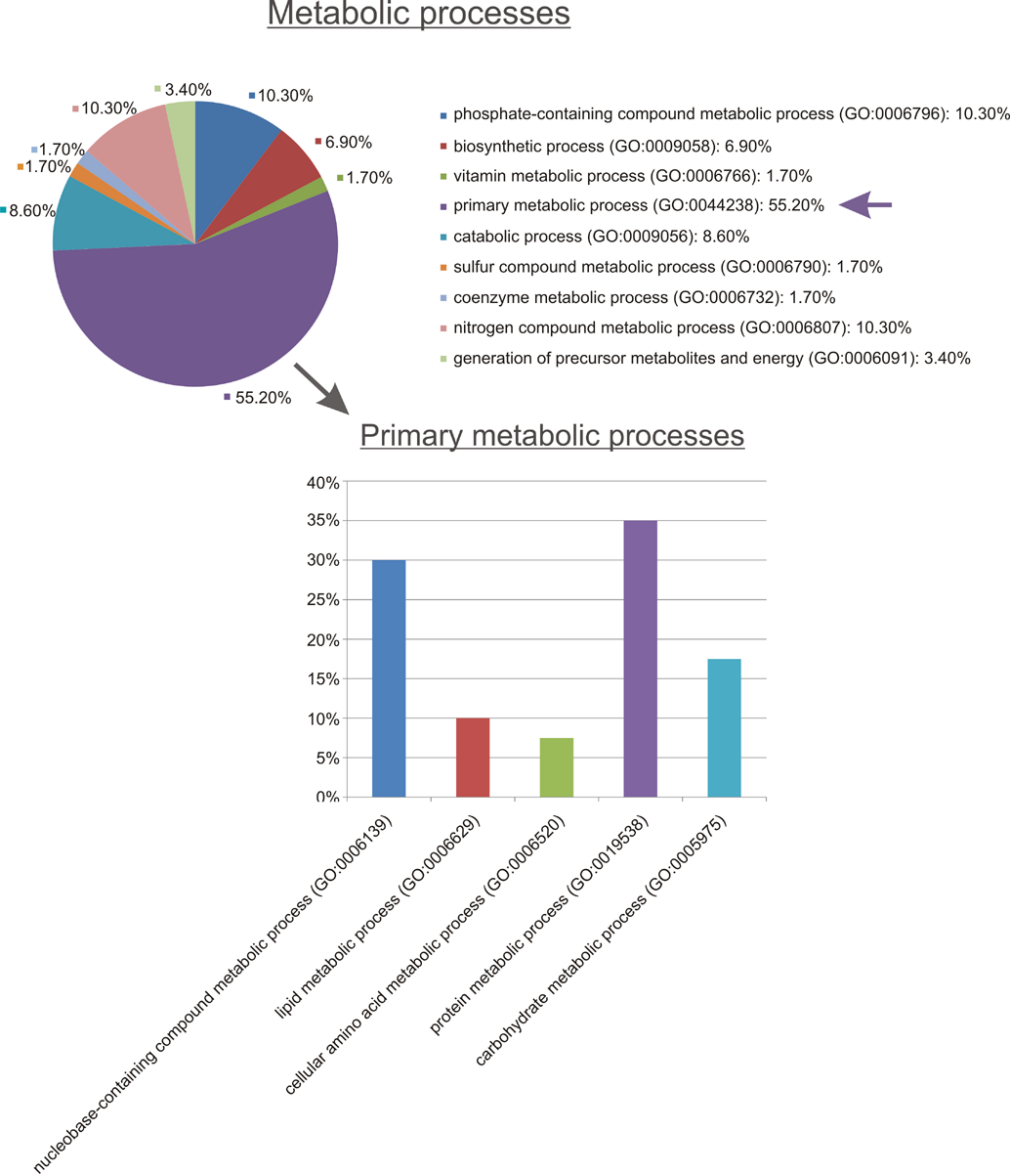
Metabolic processes related to proteins affected by DIO1 expression in ccRCC cells. Analysis was performed using PANTHER (www.pantherdb.org).

**Fig 3:**
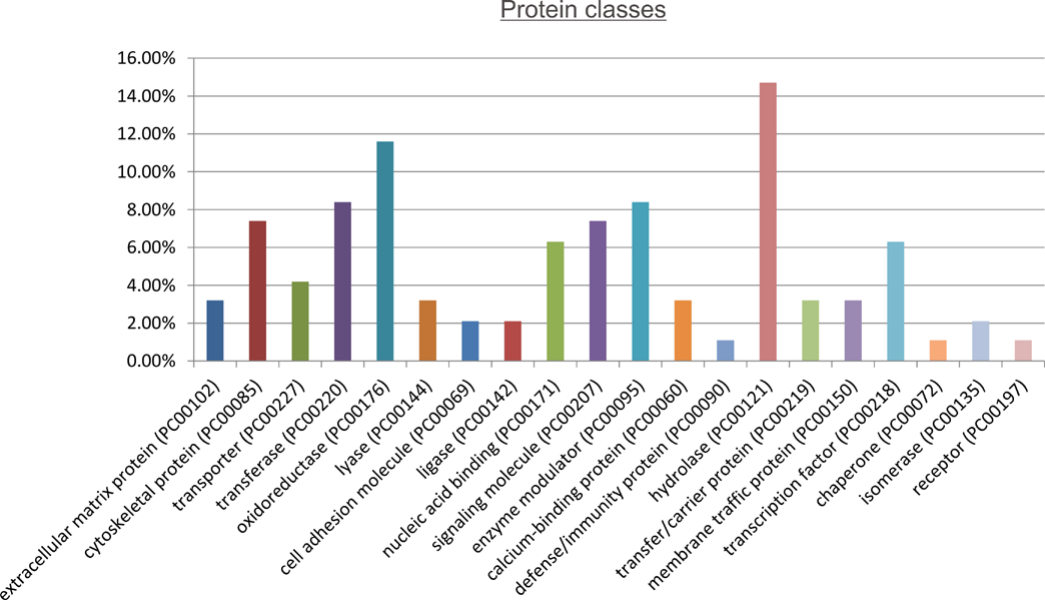
Classification of proteins that were differently expressed in DIO1(+) cells when compared with DIO1(-) cells. The graph shows results of analysis performed using PANTHER (http://pantherdb.org).

Functional analysis using PANTHER revealed that DIO1 expression affected proteins involved in key metabolic and cellular processes (Fig 1). The key affected pathways were linked with metabolism of proteins, nucleobase-containing compounds, and carbohydrates (Fig 2). Consistently, the most prominent classes of proteins affected by DIO1 expression featured activities of hydrolases, oxidoreductases and transferases (Fig 3).

To get further insight into the functions of differentially expressed proteins, functional enrichment analysis was performed separately on groups of upregulated and downregulated proteins using PANTHER. This analysis revealed striking homogeneity of both groups of proteins. GO categorization under ‘biological processes’ indicated that UPREGULATED group was enriched in proteins mainly involved in metabolic regulation, while DOWNREGULATED group was enriched in proteins involved in adhesion (Table 3). This was further confirmed by categorization under ‘molecular function’ that showed that UPREGULATED proteins were enriched in various enzymatic activities including reductases, dehydrogenases and oxidoreductases activities, while DOWNREGULATED group was characterized by cadherin binding and cell adhesion binding (Table 3). Cellular component analysis showed that UPREGULATED proteins were mainly related to extracellular exosome, extracellular vesicle, and organelle, while DOWNREGULATED group was enriched in proteins associated with lamellipodium, focal adhesion and other cell components involved in cell-cell and cell-ECM contacts (Table 3).

**Table 3:** Enrichment analysis of proteins affected by DIO1 expression in human renal cancer cells.

**BIOLOGICAL PROCESS**	
UPREGULATED PROTEINS	DOWNREGULATED PROTEINS
• farnesol catabolic process (GO:0016488) • farnesol metabolic process (GO:0016487) • sesquiterpenoid catabolic process (GO:0016107) • cellular response to jasmonic acid stimulus (GO:0071395) • response to jasmonic acid (GO:0009753) • doxorubicin metabolic process (GO:0044598) • daunorubicin metabolic process (GO:0044597) • polyketide metabolic process (GO:0030638) • aminoglycoside antibiotic metabolic process (GO:0030647) • glycoside metabolic process (GO:0016137) • progesterone metabolic process (GO:0042448) • quinone metabolic process (GO:1901661) • aromatic amino acid family catabolic process (GO:0009074) • C21-steroid hormone metabolic process (GO:0008207) • secondary metabolic process (GO:0019748) • cellular ketone metabolic process (GO:0042180) • cellular aldehyde metabolic process (GO:0006081) •cofactor metabolic process (GO:0051186) • small molecule catabolic process (GO:0044282) • carboxylic acid metabolic process (GO:0019752) • oxoacid metabolic process (GO:0043436) • organic acid metabolic process (GO:0006082) • oxidation-reduction process (GO:0055114) • small molecule metabolic process (GO:0044281) • single-organism metabolic process (GO:0044710)	• regulation of cell adhesion (GO:0030155)
**MOLECULAR FUNCTIONS**	
UPREGULATED PROTEINS	DOWNREGULATED PROTEINS
• geranylgeranyl reductase activity (GO:0045550) • indanol dehydrogenase activity (GO:0047718) • ketosteroid monooxygenase activity (GO:0047086) • phenanthrene 9,10-monooxygenase activity (GO:0018636) • trans-1,2-dihydrobenzene-1,2-diol dehydrogenase activity (GO:0047115) • androsterone dehydrogenase activity (GO:0047023) • alditol:NADP+ 1-oxidoreductase activity (GO:0004032) • alcohol dehydrogenase (NADP+) activity (GO:0008106) • aldo-keto reductase (NADP) activity (GO:0004033) • oxidoreductase activity, acting on the CH-CH group of donors, NAD or NADP as acceptor (GO:0016628) • oxidoreductase activity, acting on paired donors, with incorporation or reduction of molecular oxygen, NAD(P)H as one donor, and incorporation of one atom of oxygen (GO:0016709) • oxidoreductase activity, acting on NAD(P)H, quinone or similar compound as acceptor (GO:0016655) • oxidoreductase activity, acting on the CH-OH group of donors, NAD or NADP as acceptor (GO:0016616) • oxidoreductase activity, acting on CH-OH group of donors (GO:0016614) • monooxygenase activity (GO:0004497) • oxidoreductase activity, acting on NAD(P)H (GO:0016651) • oxidoreductase activity, acting on paired donors, with incorporation or reduction of molecular oxygen (GO:0016705) • carboxylic acid binding (GO:0031406) • organic acid binding (GO:0043177) • oxidoreductase activity (GO:0016491) • catalytic activity (GO:0003824)	• cadherin binding (GO:0045296) • cell adhesion molecule binding (GO:0050839)

**CELLULAR COMPONENT**	
UPREGULATED PROTEINS	DOWNREGULATED PROTEINS
• extracellular exosome (GO:0070062) • extracellular vesicle (GO:1903561) • extracellular organelle (GO:0043230) • extracellular region part (GO:0044421) • vesicle (GO:0031982) • extracellular region (GO:0005576)	• lamellipodium (GO:0030027) • focal adhesion (GO:0005925) • adherens junction (GO:0005912) • cell-substrate adherens junction (GO:0005924) • cell-substrate junction (GO:0030055) • anchoring junction (GO:0070161) • cell junction (GO:0030054) • cytosol (GO:0005829) • extracellular exosome (GO:0070062) • extracellular vesicle (GO:1903561) • extracellular organelle (GO:0043230) • vesicle (GO:0031982) • cytoplasmic part (GO:0044444) • cytoplasm (GO:0005737) • intracellular part (GO:0044424) • intracellular (GO:0005622)

Full data of enrichment analysis performed using http://geneontology.org/ platform and PANTHER Overrepresentation Test (release 20160715) are given in S3 Table.

**Fig 4:**
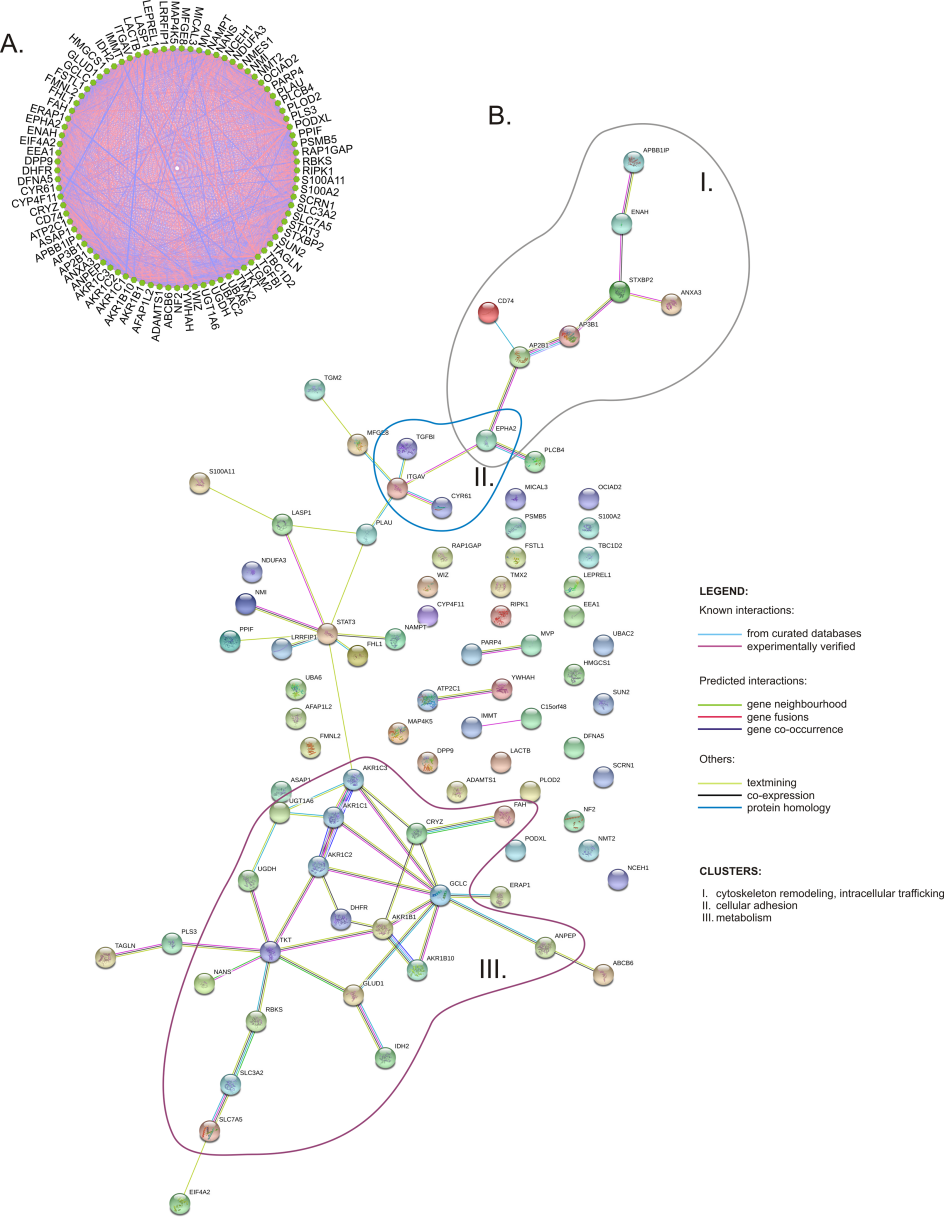
The network of proteins affected by DIO1 expression. **A.** Network of expression correlations; the proteomic data of protein expression levels were analyzed using Metscape/Cytoscape application. Blue lines: negative correlations, red lines: positive correlations. Thickness of lines indicates strength of correlations. **B.** Protein interaction network generated with STRING v. 10.5 accessed on 2017.11.06. with default settings (minimum required interaction score: medium confidence 0.4). Three major clusters are labelled as I (cytoskeleton remodeling and intracellular trafficking), II (cellular adhesion), and III (metabolism).

Next, we analyzed possible functional links between the proteins affected by DIO1 expression. Correlation matrix analysis revealed that the expressions of proteins were highly correlated (Fig 4 and S4 Table), confirming that their expressions were coordinately changed following DIO1 expression. Protein interaction network generated with STRING tool revealed three major clusters of proteins, involved in cytoskeleton remodeling and intracellular trafficking, cellular adhesion, and metabolism (Fig 4).

### Validation of proteomic analysis

**Fig 5:**
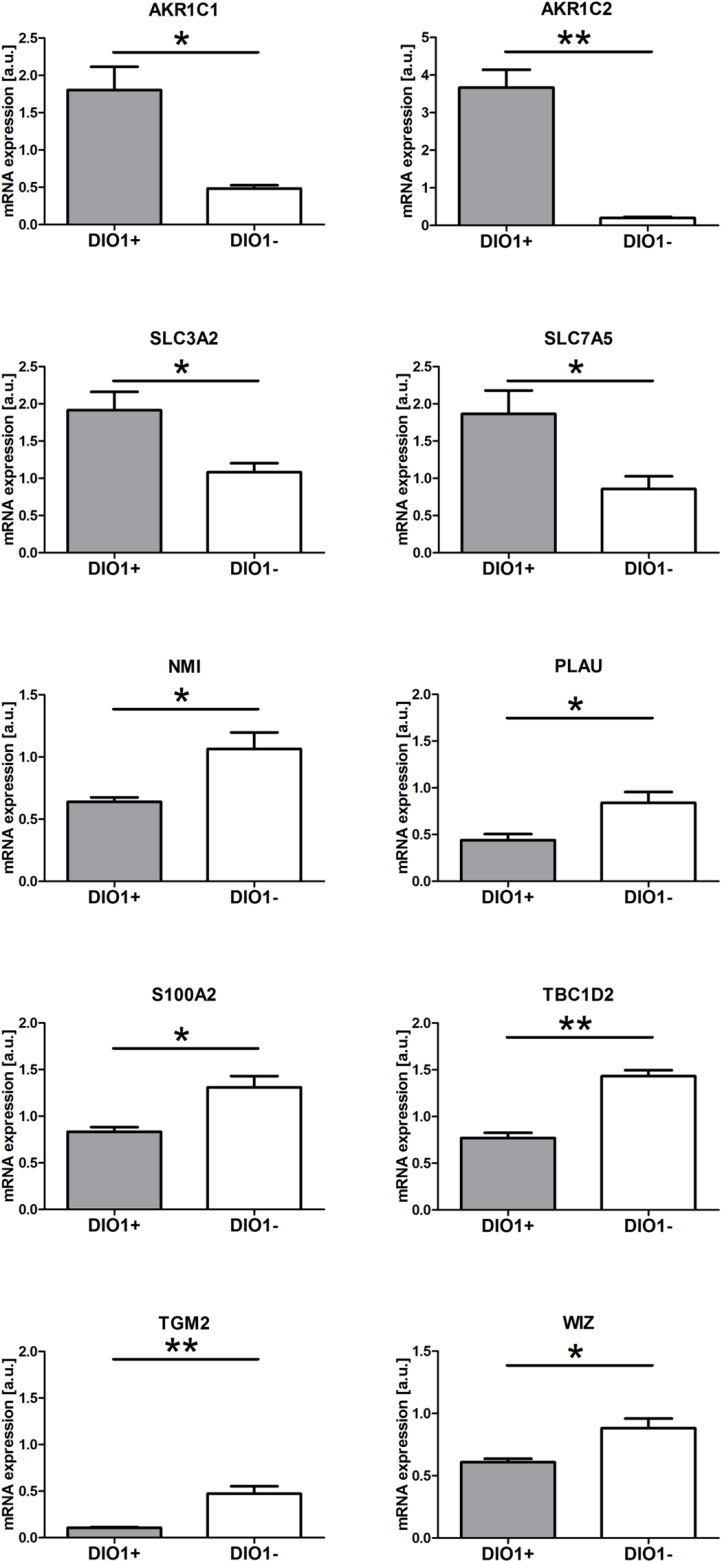
Validation of proteomic data using qPCR. The plots show mean ± SEM results of three independent biological experiments performed on KIJ265T-DIO1(+) cells when compared with KIJ265-DIO1(-) cells. Statistical analysis was performed using *t*-test. *p<0.05, **p<0.01.

To validate the results of proteomic analysis, expressions of ten genes encoding proteins affected by DIO1 expression were verified using qPCR in two RCC-derived cell lines, KIJ265T and KIJ308T, with or without ectopic expression of DIO1. The expressions of all ten genes were changed in accordance with the results of proteomic analysis (Fig 5). Specifically, the expressions of *AKR1C1*, *AKR1C2*, *SLC3A2*, and *SLC7A5* were increased in KIJ265T-DIO1(+) cells when compared with control cells (p<0.05) while expressions of *NMI*, *PLAU*, *S100A2*, *TBC1D2*, *TGM2* and *WIZ* were decreased in KIJ265T-DIO1(+) cells when compared with control cells (p<0.05) (Fig 5). Furthermore, for the vast majority of analyzed genes (except for *SLC3A2*, *SLC7A5*, and *TBC1D2*) similar trend was observed when gene expression was validated in independent RCC-derived cell line, KIJ308T, with or without ectopic expression of DIO1 (S1 and S2 Figs).

### The expressions of DIO1-affected genes correlate with DIO1 transcript concentration in renal cancer tissues

**Fig 6:**
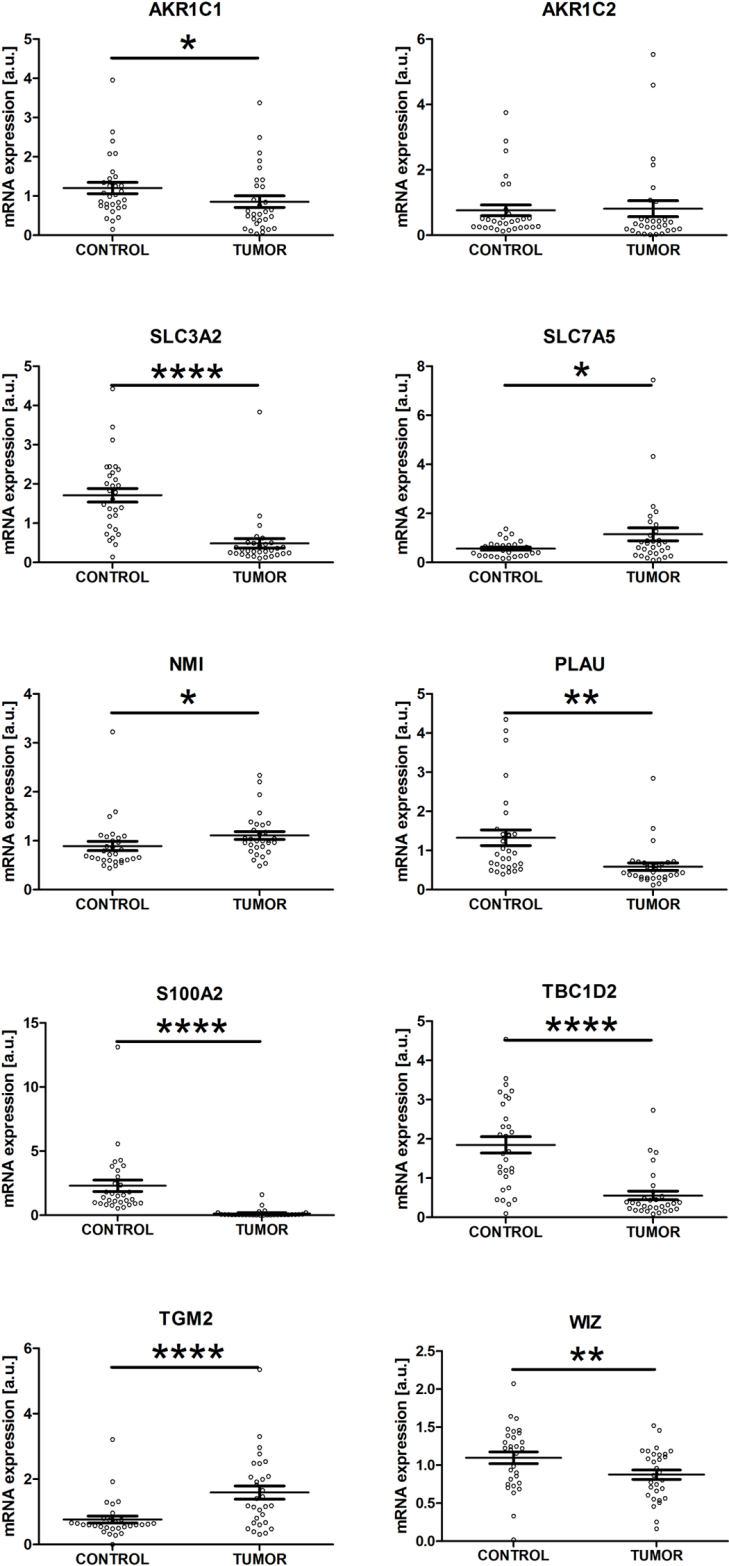
The transcript expression of genes affected by DIO1 restoration is disturbed in renal cancer. The plots show results of qPCR analysis performed in 30 matched pairs of tumor (TUMOR) and control (CONTROL) tissue samples. Statistical analysis was performed using Wilcoxon matched pairs signed test. * p<0.05; **p<0.01; **** *p*<0.0001.

To check if genes affected by DIO1 expression could be linked with altered DIO1 function in renal tumors, we analyzed their expressions in tissue samples derived from 30 patients with ccRCC. As previously reported [19,20], the expression of *DIO1* transcript was markedly decreased in renal tumors (S3 Fig). Furthermore, the expression of the vast majority of analyzed genes was altered in tumor samples when compared with control samples (Fig 6). Specifically, the transcript expressions of *AKR1C1*, *S100A2*, *PLAU*, *SLC3A2*, *TBC1D2*, and *WIZ* were decreased, while expressions of *TGM2*, *SLC7A5*, and *NMI* were increased in renal tumors when compared with non-tumorous control samples (Fig 6). The expression of *AKR1C2* was not statistically significantly changed in ccRCC tumors.

**Fig 7:**
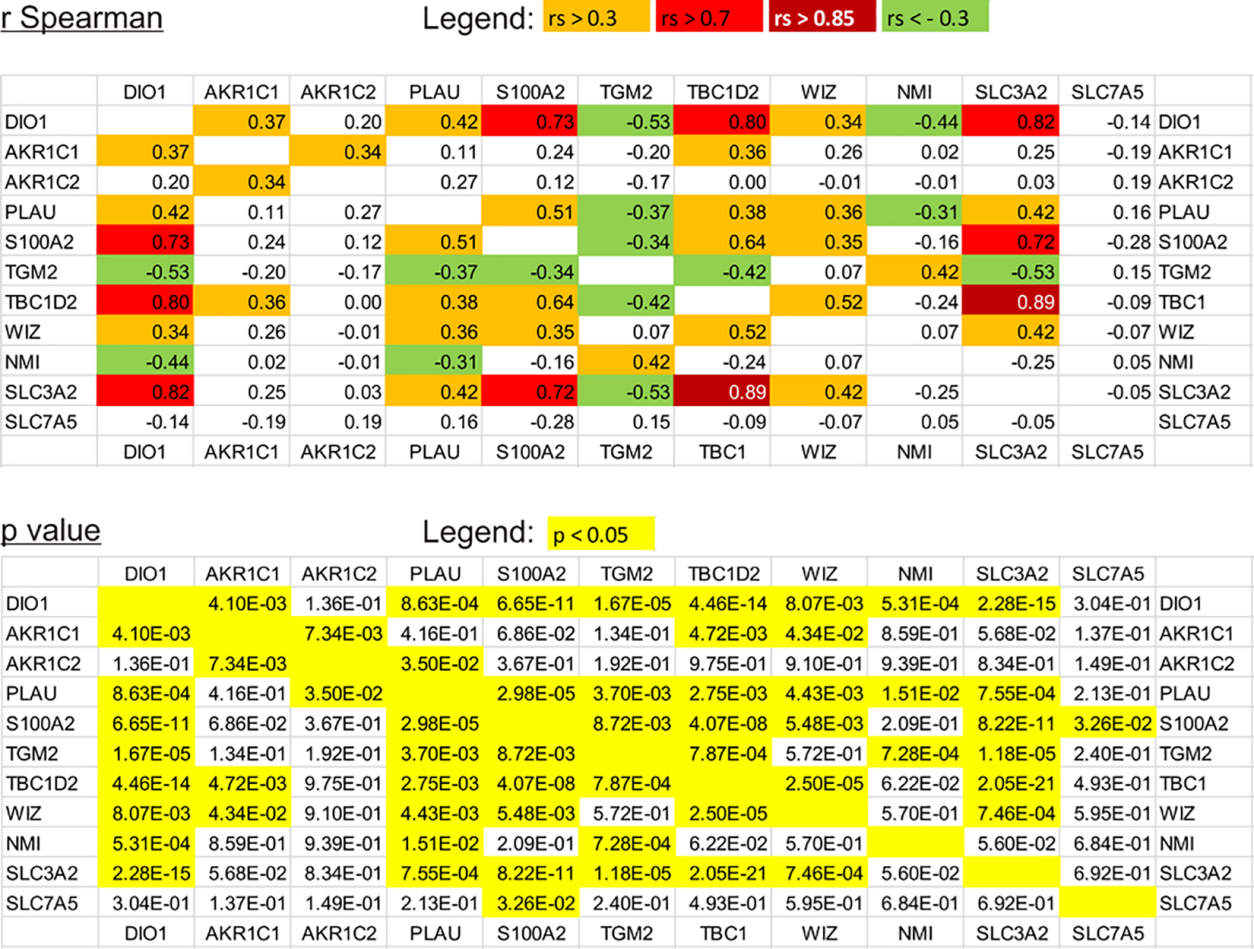
Matrix of correlations between the transcript expression of DIO1 and DIO1-affected genes in renal tumors. The upper table shows Spearman's rank correlation coefficient values for gene expressions analyzed in 30 RCC tumors and 30 paired-matched controls. Dark red: r_s_≥0.85, red: 0.85>r_s_≥0.7, orange: 0.7>r_s_≥0.3, green r_s_<-0.3. The lower table shows p values (yellow: p<0.05).

Next, we evaluated the possible correlations between the expressions of DIO1 and its affected genes. Strikingly, the transcript expressions of most genes (except for *AKR1C2* and *SLC7A5*) strongly correlated with DIO1 levels in analyzed tissue samples (Fig 7). Specifically, the expressions of *AKR1C1*, *PLAU*, *S100A2*, *TBC1D2*, *WIZ*, and *SLC3A2* were positively correlated (r Spearman ranging from 0.34 for *WIZ* to 0.82 for *SLC3A2*) while expressions of *TGM2* and *NMI* were negatively correlated with *DIO1* levels (r Spearman: -0.53, and -0.44, respectively) (Fig 7). In case of *AKR1C1*, *TGM2*, *NMI*, and *SLC3A2*, these correlations corresponded well with the direction of DIO1-induced changes of expression (Fig 5).

### Altered expression of DIO1-affected genes correlates with poor survival of ccRCC patients

**Fig 8:**
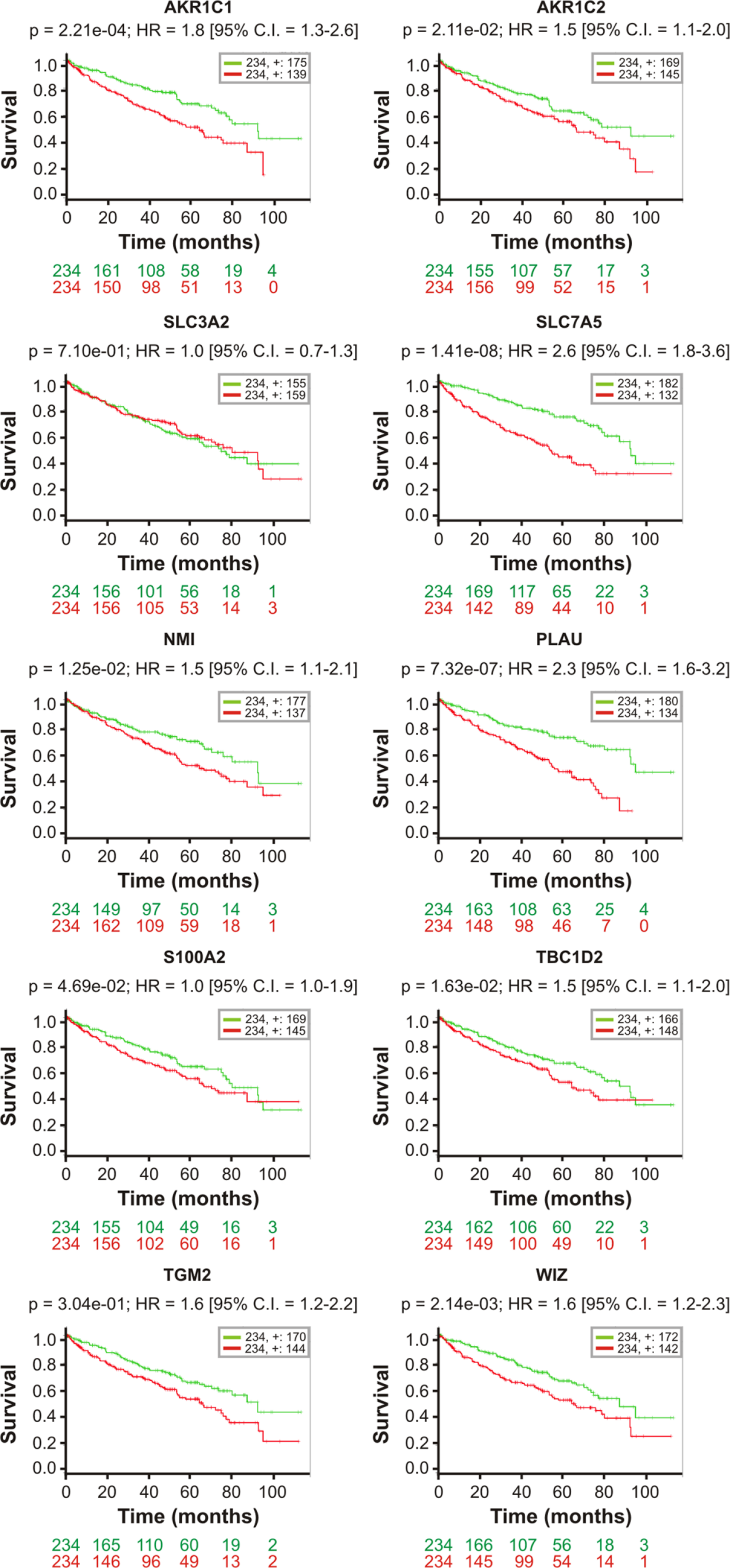
Altered transcript expression of DIO1-affected genes correlates with poor survival of renal cancer patients. Kaplan-Meyer analysis for DIO1-affected genes identified in the study. The analysis was performed on independent cohort of 468 patients with ccRCC, basing on transcriptomic data published by The Cancer Genome Atlas Network Consortium. The red and green lines depict patients with high and low risk of death, respectively. The numbers of patients in each group are shown below graphs. Censored observations are shown with +. Log-rank *p* values, hazard ratio (HR) and confidence intervals (CI) are shown above each graph. Expression of genes in each risk group is given in S4 Fig.

Next, we evaluated the possible links between the expression of DIO1-affected genes and ccRCC progression. To this end, we took advantage of the publically available data of The Cancer Genome Atlas (TCGA, [43]). This analysis, performed on transcriptomic data of 468 patients, showed that altered expressions of *AKR1C1*, *AKR1C2*, *SLC7A5*, *NMI*, *PLAU*, *TBC1D2*, *TGM2*, and *WIZ* correlated with poor survival of ccRCC patients. There was no such correlation for *SLC3A2*, while for *S100A2* the correlation with survival of patients was on the border of statistical significance (Fig 8).

### Induced DIO1 expression affects intracellular level of thyroxine

**Fig 9:**
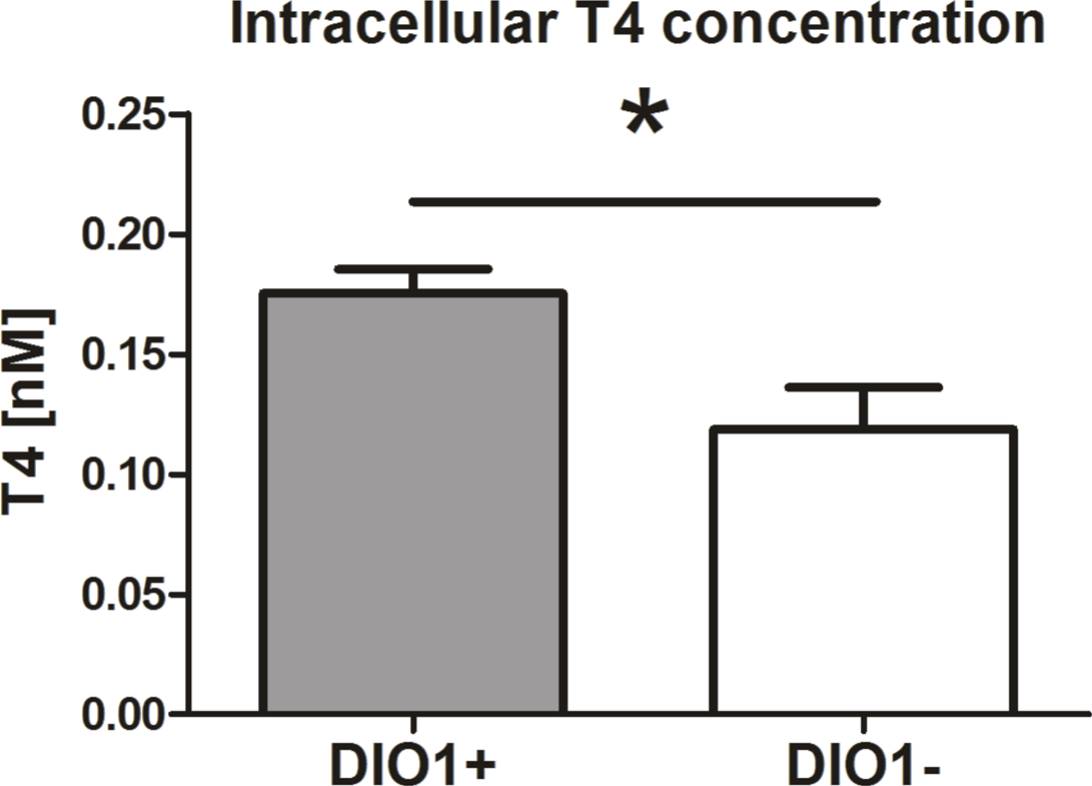
Increased T4 concentration in renal cancer cells with re-expressed DIO1. Intracellular T4 concentration in renal cancer cells with (DIO1+) or without (DIO1-) ectopic DIO1 expression. The plots show mean ± SEM results of three independent biological experiments performed on KIJ265T-DIO1(+) cells and KIJ265-DIO1(-) cells. Statistical analysis was performed using *t*-test. T3 measurements were below the detection limit. *p<0.05.

Finally, to see if the ectopic DIO1 expression influenced the levels of thyroid hormones, we measured intracellular concentrations of T4 and T3. T3 measurements were below of the detection limit. However, in agreement with enhanced transcript expression of LAT1 transporter subunits, we observed a substantial increase in cellular concentration of T4 (Fig 9).

## Discussion

To our knowledge, this is the first study addressing the effects of altered iodothyronine deiodinase expression at the proteome level. In our previous report we found that restoration of DIO1 expression in renal cancer cells inhibits their proliferation and migration [21]. Now we show that induction of DIO1 expression in renal cancer cells leads to profound changes in cellular proteome and affects the expression of genes and proteins involved in metabolic regulation, oxidative stress, autophagy and adhesion. Remarkably, altered expression of genes encoding proteins affected by DIO1 re-expression correlates with poor survival of renal cancer patients. We also demonstrate that DIO1 expression induces expression of both subunits of the thyroid hormone transporter LAT1 and increases intracellular T4 concentrations.

ccRCC is a metabolic disease [6]. The key modifications of ccRCC metabolism include Warburg effect, activation of pentose phosphate pathway (PPP), suppression of TCA cycle, and activation of lipogenesis. These changes provide cancer cells with high amounts of compounds (e.g. nucleotides, amino acids, lipids) that can serve as ‘building blocks’ for intensively proliferating cells. In our study, restoration of DIO1 expression resulted in moderate induction of enzymes involved in key pathways that undergo metabolic reprogramming in ccRCC tumors such as transketolase (TKT), nicotinamide phosphoribosyltransferase (NAMPT), and mitochondrial isoform of isocitrate dehydrogenase (IDH2). In ccRCC cells, NAMPT inhibition attenuates their growth [45]. Strikingly, and counterintuitively to the anti-tumor activity of DIO1 [21], restoration of DIO1 expression resulted in moderate increase of TKT, NAMPT and IDH2 protein levels (Table 1). DIO1 affected also expression of protein involved in amino acid metabolism (LAT1/SLC7A5 and its binding partner, CD98/4F2hc/SLC3A2), NADH oxidation (NDUFA3), and several proteins involved in lipid and steroid metabolism such as CYP4F11, AKR1C1, AKR1C2, AKR1C3, and AKR1B10. CYP4F11 catalyzes synthesis of 20-hydroxyeicosatetraenoic acid (20-HETE) which stimulates proliferation of ccRCC cells [46]. Increased expression of proteins involved in sex steroid inactivation (AKR1C1, AKR1C2, AKR1C3, AKR1B10) in DIO1 re-expressing renal cancer cells might indicate decreased local production of androgens, which have been linked to proliferation of renal and other cancers expressing androgen receptor. In particular, it was recently revealed that intracrine biosynthesis of androgen contributes to tumor growth of renal cell carcinoma [47]. Due to the low cellular concentration of nuclear hormone receptors these might escape our proteome analysis missing low abundance receptor proteins compared to classical metabolic enzymes and structural proteins.

Increased levels of TKT, NAMPT, IDH2, LAT1, NDUFA3, and CYP4F11 proteins indicate that restored DIO1 expression further boosts pro-tumorous metabolic changes in ccRCC cells. It is known that such reprogramming of cancer metabolism is tightly associated with intensified production of reactive oxygen species (ROS) [48]. For instance, it was shown that 20-HETE stimulates formation of superoxide which induces proangiogenic signaling in endothelial cells [49]. Polyunsaturated lipids that are important elements of lipoproteins and cellular membranes are particularly vulnerable targets of ROS [50]. The breakdown of lipid peroxides yields reactive bifunctional electrophiles such as 4-hydroxy-2-nonenal (4-HNE) that can further damage amino acids and DNA. Remarkably, in cells with restored DIO1 expression we observed robust induction of aldo-keto reductases (AKR1C2, AKR1B10, AKR1C1, and AKR1C3) that protect cells against toxic effects of 4-HNE [50]. The AKRs are known biomarkers of activity of Nrf2, a basic leucine zipper (bZIP) transcription factor that controls the expression of genes involved in response to oxidative stress [51]. Our further analysis revealed that a significant proportion of proteins affected by DIO1 expression was previously reported as regulated by Nrf2 (see: S4 Table and references thereof). They include GCLC that plays an essential role in synthesis of glutathione, an important antioxidant in ccRCC cells [7] or UGT1A6 that eliminates the products of oxidative metabolism via glucuronidation [52]. These results suggest that restoration of DIO1 expression in ccRCC cells may possibly result in ROS generation that in turn triggers compensatory mechanisms that protect against oxidative stress. While moderate ROS production can stimulate proliferation, their excess leads to oxidative damage of DNA, proteins and lipids. To avoid cell death induced by oxidative stress, cancer cells need to carefully maintain the balance between ROS generation and scavenging [48]. Strikingly, induced DIO1 expression resulted also in initiation of mechanisms that can mitigate the ROS scavenging activity. Mitochondrial glutamate dehydrogenase GLUD1 controls redox homeostasis in cancer cells via the product of its activity, α-ketoglutarate, and its metabolite, fumarate [53]. The latter binds to the key ROS-scavenging enzyme, glutathione peroxidase (GPx), leading to its activation, thereby providing highly efficient protection against oxidative stress. Suppression of GLUD1 activity leads to decreased fumarate levels and GPx activity, elevation of ROS and attenuation of cancer cell proliferation and tumor growth [53]. In our study, DIO1 expression downregulated GLUD1 with concomitant increase of levels of fumarylacetoacetase (FAH) which catalyzes hydrolysis of fumarylacetoacetate to fumarate and acetate. This data suggests that while on one hand DIO1 expression results in induction of proteins involved in antioxidative protection, on the other hand it can also potentially attenuate the activity of the key ROS-scavanger, GPx.

The fact that restoration of DIO1 expression in ccRCC cells leads to attenuation of proliferation and migration [21] indicates that anti-oxidative response observed in this study could be inefficient and that oxidative stress generated by DIO1-induced acceleration of pro-tumorous metabolism could exceed antioxidative capacities of ccRCC cells, initiating mechanisms leading to cell death. Indeed, we observed profound reduction of NMT2, a negative regulator of apoptotic responses. NMT2 catalyzes N-myristoylation of multiple proteins and its depletion in cancer cells results in dramatic increase of apoptosis [54]. Furthermore, DIO1 induced the expression of PPIF (cyclophilin D), one of the proteins of the mitochondrial permeability transition pore. In RCC tumors, the expression of PPIF is reduced while its induction results in apoptosis and necrosis [55].

DIO1 strongly repressed the expression of proteins involved in ccRCC progression such as aminopeptidase N (ANPEP), CYR61, and TGM2. In renal tumors, enhanced ANPEP expression correlates with poor survival of patients [56], while high CYR61 levels stimulate proangiogenic activity of cancer cells [57]. Increased expression of TGM2 (transglutaminase-2) in ccRCC tumors contributes to cancerous proliferation, migration and invasion [58] and enables survival of RCC cells by a mechanism involving autophagy-dependent p53 degradation [59]. Inhibition of TGM2 activity attenuates growth of RCC xenografts in mice [60]. In our study, DIO1 expression led to a substantial downregulation of TGM2 as well as TBC1D2 and NMI, two other regulators of autophagy. These results raise an interesting hypothesis that DIO1 expression may interfere with autophagy mechanisms by local provision of T3 known to be involved in regulation of autophagy either directly or via acting as ligand for the T3 receptor [61,62]. In accordance with our recent study [21], DIO1 expression resulted also in suppression of TGFBI, and several other proteins (e.g. PODXL, CD74) that promote RCC progression [63–65] or act as oncogenic proteins in other cancers (e.g. FMNL2 [66], APBB1IP [67], ASAP1 [68,69], and LRRFIP1 [70,71]). Thus, it may be concluded that DIO1 re-expression in ccRCC cells results in global downregulation of proteins that directly promote cancerous proliferation, migration and invasion. Additional information on protein of which expression was affected by DIO1 is given in Supplementary S1 File.

Regarding the mechanisms that mediate DIO1 effects in RCC cells, several scenarios can be considered. The genes encoding proteins affected by DIO1 expression may be regulated by the product of DIO1 activity, T3. Indeed, we observed a pronounced induction of the SLC7A5, a T3 early-response gene [72]. Remarkably, together with SLC3A2, SLC7A5 forms an efficient transporter facilitating intracellular trafficking of thyroid hormones [45]. In agreement with induced expression of SLC7A5 and SLC3A2, we observed substantial increase in intracellular T4 concentrations. Unfortunately, we could not detect T3 in these cells but our recent study suggested that DIO1 ectopically expressed in renal cancer cells deiodinates T4 to produce T3 that induces changes in expression of target genes [21]. Specifically, we showed that that supplementation of KIJ265T and KIJ308T cells (not transfected with DIO1-expressing plasmids) with T3 did not recapitulate the effects of ectopic DIO1 expression, suggesting inefficient intracellular transport of T3. These results were in accordance with data showing decreased expression of T3 transporters (including SLC3A2) in renal tumors. In contrast, supplementation of KIJ265T-DIO1(+) cells with DIO1 substrate, T4, resulted in changed expression of DIO1-affected genes in a dose dependent manner. Remarkably, no effects of T4 supplementation were observed in RCC cells devoid of ectopic DIO1 expression. These results indicated that ectopic DIO1 expression enabled conversion of T4 to T3 which could further affect the expression of target genes [21]. DIO1 may also affect gene expression by indirect T3 actions. Thyroid hormone is a powerful inducer of oxidative stress [62], stimulates rapid translocation of Nrf2 from cytosol to nucleus [73], and activates the expression of Nrf2 targets, AKR1C1-C3 [74] and TKT [75]. Thus, the robust DIO1-induced upregulation of genes which are known Nrf2 targets (S5 Table and references thereof) may possible be explained by T3-mediated regulation of Nrf2. Furthermore, apart from iodothyronines, the product of DIO1 activity is the iodide. Treatment of breast cancer cells with iodine/iodide results in upregulation of ACR1C1 and SLC7A5 [76], the two genes that were upregulated by DIO1 overexpression in our study.

In general, the results of our study are in agreement with the known effects of deiodinases in cancer and healthy cells. The changes in expression of iodothyronine deiodinases provide tight spatio-temporal control of cellular T3 levels that initiate signaling essential for proliferation and differentiation. Upregulation of type 3 deiodinase (DIO3), a T3 inactivating enzyme, is required during initial, proliferative phase of myogenesis. In contrast, expression of DIO2 at late phase of myogenesis enables T3-mediated signaling that stimulate cell differentiation [77]. Similarly, in proliferating colorectal cancer stem cells (CR-CSCs), the expression of DIO2 is attenuated, while its expression rapidly increases during cell differentiation. In contrast, DIO3 expression is oppositely regulated during CR-CSCs’ differentiation [78]. Most of described so far T3 effects on cellular proliferation and differentiation are mediated by its specific nuclear receptors (TRs) [12]. In our proteomic analysis, we could not detect TRs, as well as DIO2 and DIO3 which can result from low abundance of these proteins in renal cancer [20,21,79]. Since genes encoding TRs are T3-regulated, it can be expected that their expression could be enhanced by DIO1 re-expression. Remarkably, thyroid hormones can also influence cell functioning by various non-genomic mechanisms that do not necessarily involve TRs [80], providing another possible explanation for deiodinase effects in cancer cells. We found that DIO1-induced expression changes detected by proteomic and qPCR analysis were highly consistent. The mRNA expression changes of all ten validated genes were in accordance with the results of proteomic analysis. This may possibly suggest that DIO1 re-expression may result in initiation of transcriptional reprograming. Interestingly, DIO1 expression resulted in decreased levels of transcriptional regulators, STAT3 and WIZ. STAT3 is a well-known regulator of multiple genes involved in apoptosis, proliferation, migration and invasion [81]. Its persistent activation in cancers, including ccRCC, directly contributes to tumor development and progression [81,82]. Inhibition of STAT3 pathway in ccRCC induces apoptosis, attenuates angiogenesis and metastasis in renal cancer mouse models [83,84]. DIO1 effects could be also mediated by WIZ, a transcriptional co-regulator, involved in chromatin regulation [85] or EIF4A2, an important regulator of translation [86]. Future studies are needed to reveal the detailed mechanism of DIO1-induced changes in proteome of renal cancer cells.

While interpreting the results of our study, it should be also considered that DIO1-induced proteome changes were analyzed in a ccRCC-derived cell line. In contrast, tumors are a mixture of different types of cells, including these of epithelial, endothelial or stromal origin. The remarkable correlations between the expression of DIO1 transcript and some of DIO1-affected genes in tissue samples derived from ccRCC patients may possibly suggest that loss of DIO1 expression in ccRCC tumors may influence the pattern of gene expression in vivo. It would be interesting to see if changes in DIO1 expression may affect other cells and processes that contribute to tumor formation, in particular angiogenesis or infiltrating cells of immune system. These aspects of DIO1 activity in cancer cells should be investigated in the future.

## Conclusions

**Fig 10:**
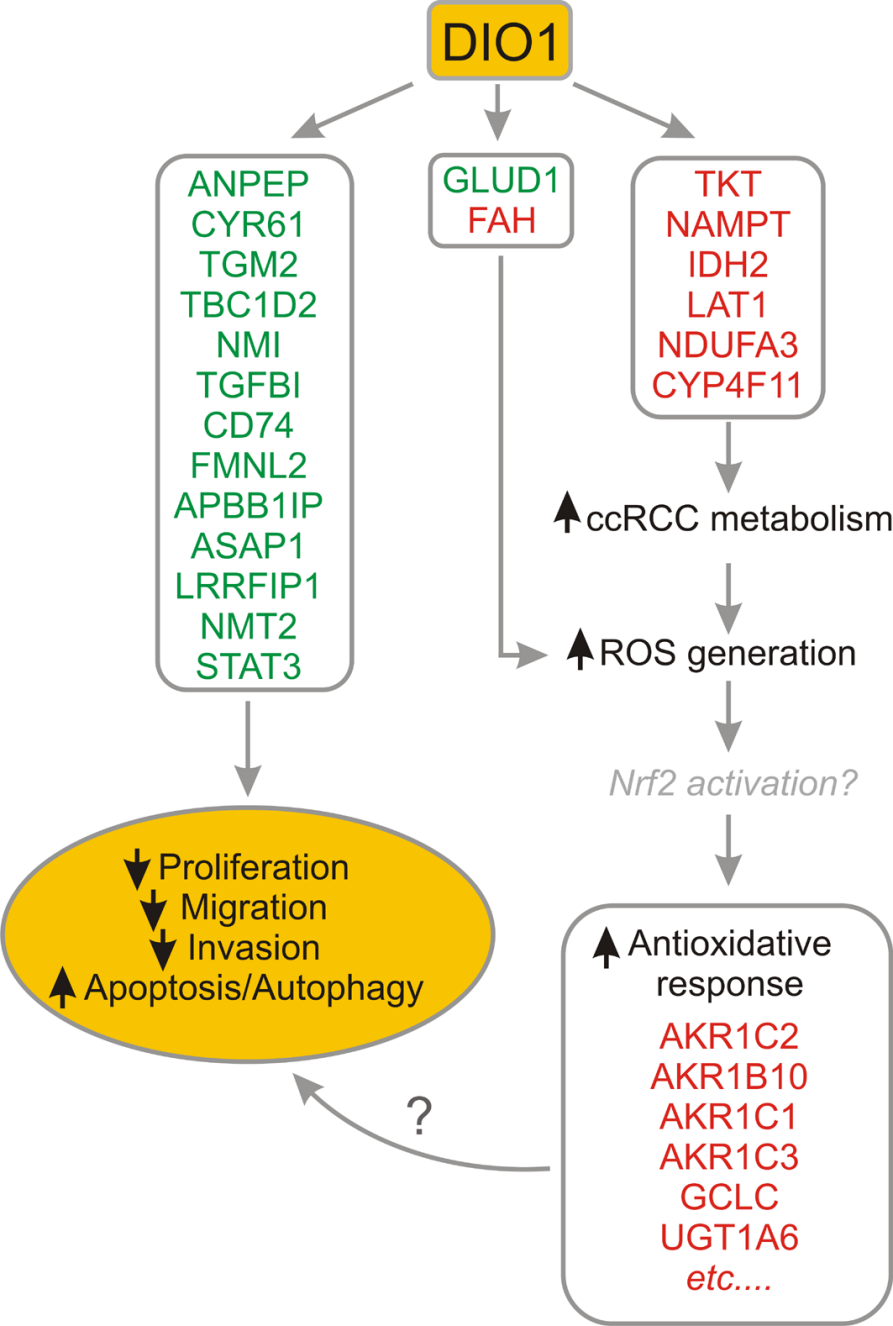
The model depicting the proteomic effects of DIO1 restoration in renal cancer. Changes in protein levels are illustrated with green (decreased level) and red (increased level) colors. Induction of DIO1 expression in renal cancer cells results in robust downregulation of oncoproteins that are well known inhibitors of apoptosis and promoters of ccRCC proliferation, migration and invasion (left side of the drawing). Simultaneously, restoration of DIO1 in ccRCC cells leads to enhanced expression of proteins that contribute to metabolic reprogramming of renal tumors and affect PPP, TCA cycle, metabolism of amino acids and lipids. This may be associated with prominent induction of ROS that in turn trigger antioxidative response and results in enhanced levels of proteins of Nrf2 pathway (right side of the drawing). On the other hand, induced DIO1 expression can also possibly result in attenuation of ROS-scavenging system by decreasing GLUD1 and inducing FAH (middle part of the drawing). Altogether, this may possibly result in ROS levels that exceed the compensatory buffering systems of ccRCC cells and trigger mechanisms leading to apoptosis or autophagy.

In summary, the results of this and our previous [21] study suggest that restoration of DIO1 expression affects functioning of cancer cells in two modes (Fig 10). On one hand, it further boosts protumorous changes in ccRCC metabolism, by changing the levels of proteins involved in PPP, TCA cycle or metabolism of lipids, steroids and amino acids. This in turn may possibly result in massive generation of ROS that initiate robust induction of proteins involved in antioxidative response. If ROS generation exceeds the buffering capacities of anti-oxidative system, this may initiate apoptotic responses. On the other hand, increased DIO1 and resulting local T3 production activity may globally downregulate oncogenic proteins that promote cancerous proliferation, migration and invasion of ccRCC cells. Altogether, these changes result in suppression of cancerous phenotype of ccRCC cells with restored DIO1 expression. At the same time, the results of our study may possibly suggest that loss of DIO1 expression in ccRCC tumors could be an adaptive mechanism, protecting the cells against overstimulation of cancer metabolism and induction of autophagy and or apoptosis.

## Supporting information

S1 Fig Expression of DIO1 protein in KIJ265T and KIJ308T cells following stable transfection with pcDNA3-DIO1 (DIO1+) or empty plasmid (DIO1-). 60 μg of protein was resolved on SDS-PAGE, β-actin was used as loading control.
(TIF)

S2 Fig The expression of DIO1-affected genes in KIJ308T-DIO1(+) and KIJ308T-DIO1(-) cells. The plots show mean ± SEM results of qPCR analysis performed in three independent biological experiments. Statistical analysis was performed using *t*-test. *p<0.05, **p<0.01. Induction of DIO1 expression in KIJ308T cells is shown in Supplementary S1 Fig.
(TIF)

S3 Fig The expression of DIO1 in renal cancer. The plots show results of qPCR analysis performed in 30 matched pairs of tumor (TUMOR) and control (CONTROL) tissue samples. Statistical analysis was performed using Wilcoxon matched pairs signed test. **** *p*<0.0001.
(TIF)

S4 Fig The expression of DIO1-affected genes stratified by risk groups. The data was retrieved from TCGA and the analysis was performed using SurvExpress; t-test was used to compute p values. p<0.05 was considered statistically significant. P values are shown above box plots for each gene. Green: expression in low risk group. Red: expression in high risk group. Note that the scales are different.
(TIF)

S1 Table Primers and probes used for qPCR. (DOC)

S2 Table Raw data of proteomic analysis. (XLSX)

S3 Table Results of enrichment analysis performed using http://geneontology.org/ platform and PANTHER Overrepresentation Test (release 20160715). (XLSX)

S4 Table Matrix of correlations between the expressions of proteins affected by DIO1 expression in renal cancer cells. The analysis was performed using GraphPad Prism 5.0. Pearson correlation coefficients were calculated on log2 normalized data.
(XLSX)

S5 Table NRF2-targets affected by DIO1 expression. ND: no data.
(DOC)

S1 File Additional information on proteins of which expression was affected by DIO1 in renal cancer cells. (DOCX)
