# Supplementary figures and images for: Restoration of type 1 iodothyronine deiodinase expression in renal cancer cells downregulates oncoproteins and affects key metabolic pathways as well as anti-oxidative system
Source: PLoS One. 2017 Dec 22;12(12):e0190179. doi: 10.1371/journal.pone.0190179 (PMC5741248; doi:10.1371/journal.pone.0190179)

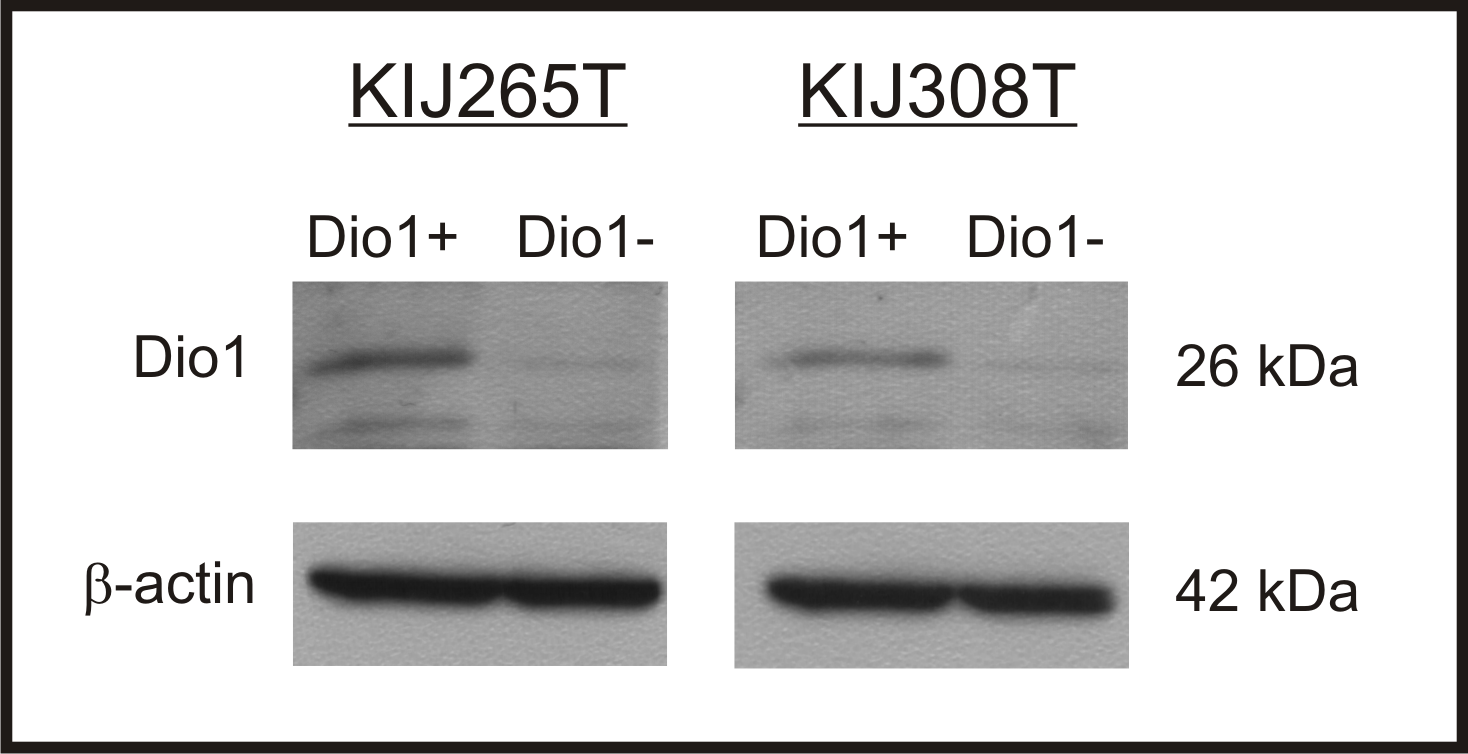

Supplement: S1 Fig — 60 μg of protein was resolved on SDS-PAGE, β-actin was used as loading control. (TIF) [file pone.0190179.s001.TIF]

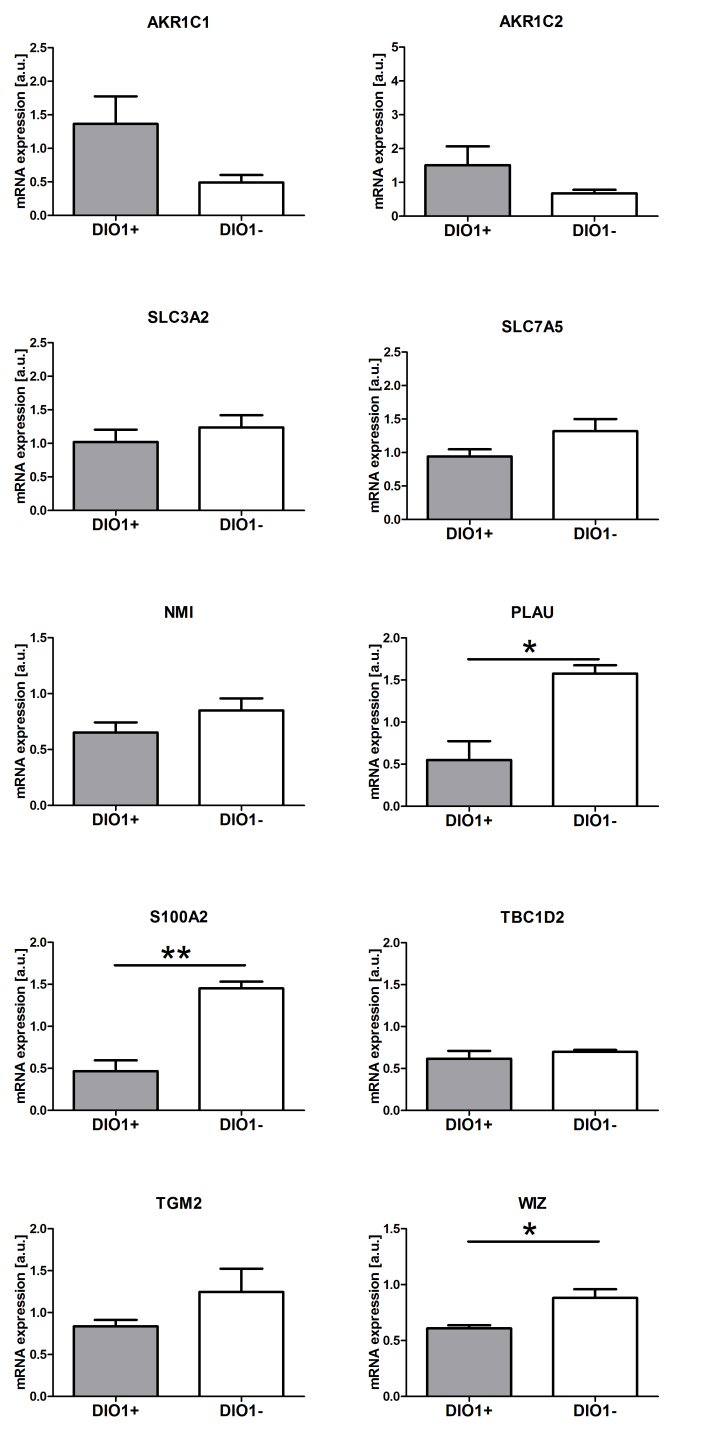

Supplement: S2 Fig — The plots show mean ± SEM results of qPCR analysis performed in three independent biological experiments. Statistical analysis was performed using t-test. *p<0.05, **p<0.01. Induction of DIO1 expression in KIJ308T cells is shown in Supplementary S1 Fig. (TIF) [file pone.0190179.s002.TIF]

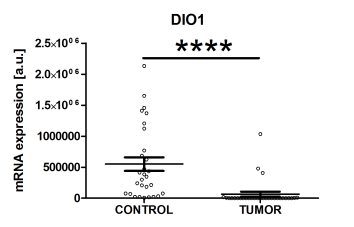

Supplement: S3 Fig — The plots show results of qPCR analysis performed in 30 matched pairs of tumor (TUMOR) and control (CONTROL) tissue samples. Statistical analysis was performed using Wilcoxon matched pairs signed test. **** p<0.0001. (TIF) [file pone.0190179.s003.TIF]

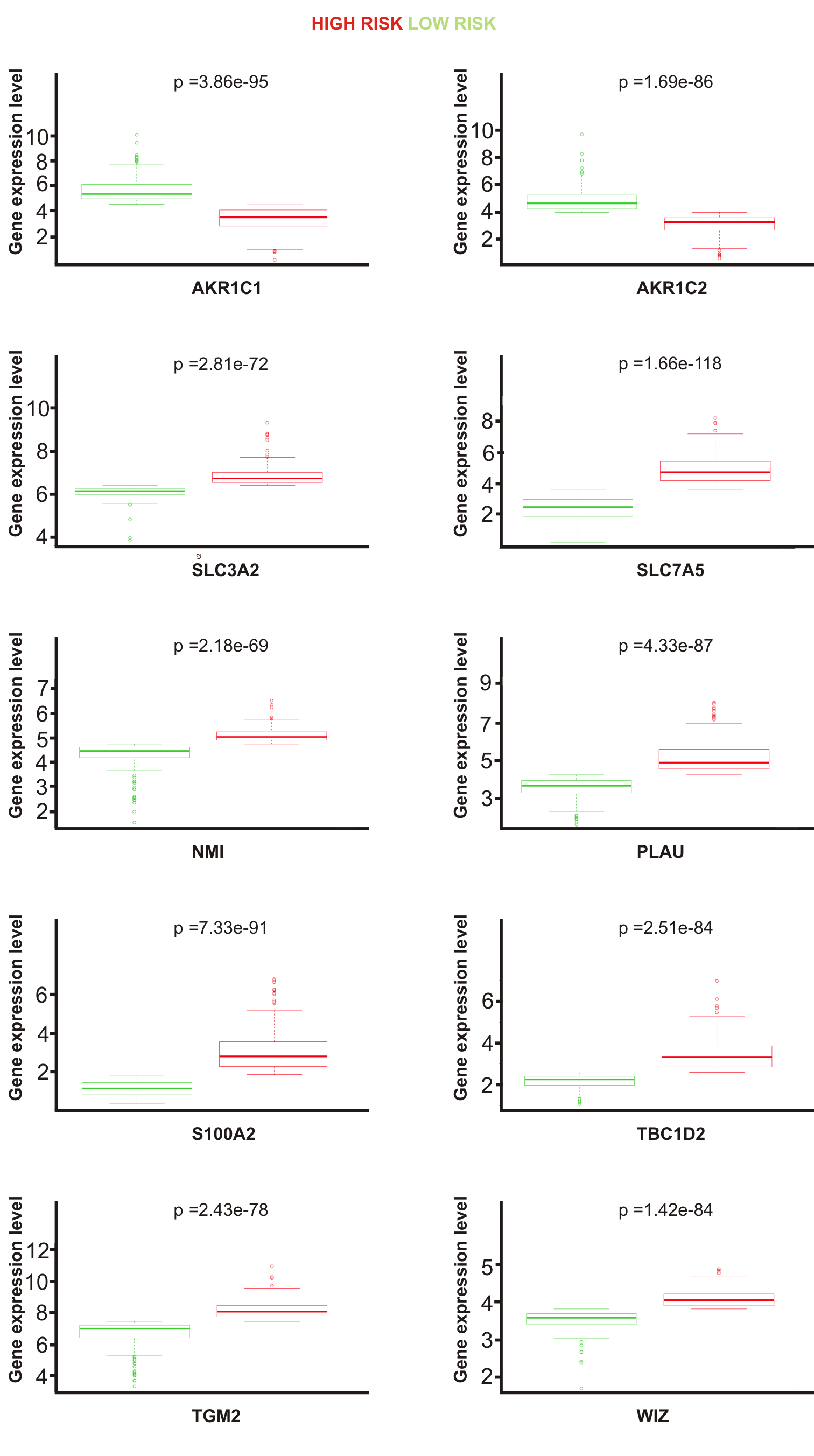

Supplement: S4 Fig — The data was retrieved from TCGA and the analysis was performed using SurvExpress; t-test was used to compute p values. p<0.05 was considered statistically significant. P values are shown above box plots for each gene. Green: expression in low risk group. Red: expression in high risk group. Note that the scales are different. (TIF) [file pone.0190179.s004.TIF]
