# Supplementary material for: Restoration of type 1 iodothyronine deiodinase expression in renal cancer cells downregulates oncoproteins and affects key metabolic pathways as well as anti-oxidative system
Source: PLoS One. 2017 Dec 22;12(12):e0190179. doi: 10.1371/journal.pone.0190179 (PMC5741248; doi:10.1371/journal.pone.0190179)
Supplement: S1 Table — (DOC) [file pone.0190179.s005.doc]

**Supplementary Table S1. Primers and probes used for Qpcr**

| ***PRIMERS*** | | | |
| --- | --- | --- | --- |
| ***Gene target*** | ***Forward primer*** | ***Reverse primer*** | ***Ref.*** |
| *AKR1C1* | h-AKR1C1-F:AGAAAGAAACATTTGCCAG | h-AKR1C1-R:GCTTCAATTGCCAATTTGGTG | This study |
| *AKR1C2* | h-AKR1C2-F:TGATGGTCACTTCATGCCT | h-AKR1C2-R:CTTCTATTGCCAATTTGACGG | This study |
| *NMI* | h-NMI-F:AATTCCTGACACATTGCGTGA | h-NMI-R:CCTCTCCGCCTCCATTTCG | This study |
| *PLAU* | h-PLAU-F:AACTGCCCAAAGAAATTCGG | h-PLAU-R:TCGGTAAAAGTGACCATTCCC | This study |
| *S100A2* | h-S100A2-F:GCTGTGCTGGTCACTACCTT | h-S100A2-R:TTCAGCCCCTCCTCATCCAC | This study |
| *SLC3A2* | h-SLC3A2-F:ACTCTTCTCCTATATCCGCCACT | h-SLC3A2-R:CCCACATCCCCAAAGTTAAGCAC | This study |
| *SLC7A5* | h-SLC7A5-F:TGTACGTGCTGACCAACCTG | h-SLC7A5-R:GTGATAGTTCCCGAAGTCCAC | This study |
| *TBC1D2* | h-TBC1-F:GATCTCTCCCTCGTCACCTTC | h-TBC1-R:CCAAGGCATAGCGAAACACCA | This study |
| *TGM2* | h-TGM2-F:ACTTCATTTTGCTCTTCAACGC | h-TGM2-R:TCTTCCGAGTCCAGGTACACA | This study |
| *WIZ* | h-WIZ-F:CGGACCTTCACATCTCACCCTTG | h-WIZ-R:CCGCTTGATCTCCACCCGTA | This study |
| *HPRT1* | HPRT1ex2RTU:TGGCGTCGTGATTAGTGATG | HPRT1ex3RTL:CAGAGGGCTACAATGTGATG | Koronowicz et al., 2016 |
| *RNA18S5* | 18sRNA-F:GTAACCCGTTGAACCCCATT | 18sRNA-R: CCATCCAATCGGTAGTAGCG | Piekielko-Witkowska et al., 2010 |
| *ACTB* | ACTB-ex3-RT-U: CGGCATCGTCACCAACTG | ACTB-ex4-RT-L: GCTGGGGTGTTGAAGGTCTC | Master et al., 2010 |
| ***PROBES*** | | | |
| ***Gene target*** | ***Probe number*** | ***Manufacturer*** | |
| *DIO1* | *Hs001174944_m1* | Thermo Fisher Scientific, Rockford, IL, USA | |
| *HPRT1* | *Hs02800695_m1* | Thermo Fisher Scientific, Rockford, IL, USA | |
| *RNA18S5* | *Hs03928985_g1* | Thermo Fisher Scientific, Rockford, IL, USA | |

**References for primers:**

Koronowicz AA, Kopeć A, Master A, Smoleń S, Piątkowska E, Bieżanowska-Kopeć R, Ledwożyw-Smoleń I, Skoczylas Ł, Rakoczy R, Leszczyńska T et al. 2016 Transcriptome Profiling of Caco-2 Cancer Cell Line following Treatment with Extracts from Iodine-Biofortified Lettuce (Lactuca sativa L.). *PLoS One* **11** e0147336 (doi: 10.1371/journal.pone.0147336. eCollection 2016)

Master A, Wójcicka A, Piekiełko-Witkowska A, Bogusławska J, Popławski P, Tański Z, Darras VM, Williams GR & Nauman A. 2010 Untranslated regions of thyroid hormone receptor beta 1 mRNA are impaired in human clear cell renal cell carcinoma. *Biochimica et Biophysica Acta (BBA) - Molecular Basis of Disease* **1802** 995-1005.

Piekielko-Witkowska A, Wiszomirska H, Wojcicka A, Poplawski P, Boguslawska J, Tanski Z & Nauman A. 2010 Disturbed expression of splicing factors in renal cancer affects alternative splicing of apoptosis regulators, oncogenes, and tumor suppressors. *PLoS One* **5** e13690
