# Supplementary material for: Restoration of type 1 iodothyronine deiodinase expression in renal cancer cells downregulates oncoproteins and affects key metabolic pathways as well as anti-oxidative system
Source: PLoS One. 2017 Dec 22;12(12):e0190179. doi: 10.1371/journal.pone.0190179 (PMC5741248; doi:10.1371/journal.pone.0190179)
Supplement: S5 Table — ND: no data. (DOC) [file pone.0190179.s009.doc]

**Supplementary Table S4**. NRF2-targets affected by DIO1 expression. ND: no data

| **Gene name** | **Protein name** | **Nrf2 regulated gene (+ stimulation; - inhibition)** | **Ref.** |
| --- | --- | --- | --- |
| *AKR1C2* | Aldo-keto reductase family 1 member C2 | + | MacLeod et al. 2009 |
| *RAP1GAP* | Rap1 GTPase-activating protein 1 | + | Lacher and Slattery, 2016 |
| *AKR1B10* | Aldo-keto reductase family 1 member B10 | + | MacLeod et al. 2009, Ooi et al., 2011 |
| *PPIF* | Peptidyl-prolyl cis-trans isomerase F, mitochondrial | ND | ND |
| *ABCB6* | ATP-binding cassette sub-family B member 6, mitochondrial | + | Lacher and Slattery, 2016 |
| *AKR1C1* | Aldo-keto reductase family 1 member C1 | + | MacLeod et al. 2009 |
| *SLC7A5* | Large neutral amino acids transporter small subunit 1 | ND | ND |
| *TAGLN* | Transgelin | ND | ND |
| *AKR1C3* | Aldo-keto reductase family 1 member C3 | + | MacLeod et al. 2009 |
| *CYP4F11* | Phylloquinone omega-hydroxylase CYP4F11 | + | Lacher and Slattery, 2016 |
| *UBAC2* | Ubiquitin-associated domain-containing protein 2 | ND | ND |
| *FSTL1* | Follistatin-related protein 1 | ND | ND |
| *MFGE8* | Lactadherin; Lactadherin short form; Medin | + | Lastres-Becker et al. 2014 |
| *FAH* | Fumarylacetoacetase | ND | ND |
| *UGT1A6* | UDP-glucuronosyltransferase 1-6 | + | Yeager et al., 2009 |
| *GCLC* | Glutamate-cysteine ligase catalytic subunit | + | Yang et al. 2005 |
| *UGDH* | UDP-glucose 6-dehydrogenase | + | Wu et al. 2012 |
| *PSMB5* | Proteasome subunit beta type-5 | + | Mi-Kyoung et al. 2003 |
| *EIF4A2* | Eukaryotic initiation factor 4A-II; Eukaryotic initiation factor 4A-II, N-terminally processed | ND | ND |
| *SLC3A2* | 4F2 cell-surface antigen heavy chain | + | Chorley et al. 2012 |
| *NAMPT* | Nicotinamide phosphoribosyltransferase | + | Shelton et al.2015 |
| *TKT* | Transketolase | + | Dinkova-Kostova and Abramov; 2015. |
| *PLOD2* | Procollagen-lysine,2-oxoglutarate 5-dioxygenase 2 | ND | ND |
| *IDH2* | Isocitrate dehydrogenase [NADP], mitochondrial | ND | ND |
| *TMX2* | Thioredoxin-related transmembrane protein 2 | ND | ND |
| *CRYZ* | Quinone oxidoreductase | + | Shelton et al., 2015 |
| *AFAP1L2* | Actin filament-associated protein 1-like 2 | ND | ND |
| *ANPEP* | Aminopeptidase N | - | Osburn et al. 2008 |
| *CYR61* | Protein CYR61 | ND | ND |
| *NMT2* | Glycylpeptide N-tetradecanoyltransferase 2; Glycylpeptide N-tetradecanoyltransferase | ND | ND |
| *MICAL3* | Protein-methionine sulfoxide oxidase MICAL3 | ND | ND |
| *PLAU* | Urokinase-type plasminogen activator; Urokinase-type plasminogen activator long chain A; Urokinase type plasminogen activator short chain A; Urokinase-type plasminogen activator chain B | ND | ND |
| *WIZ* | Protein Wiz | ND | ND |
| *ANXA3* | Annexin A3; Annexin | ND | ND |
| *PLCB4* | 1-phosphatidylinositol 4,5-bisphosphate phosphodiesterase beta-4 | ND | ND |
| *FMNL2* | Formin-like protein 2 | ND | ND |
| *FHL1* | Four and a half LIM domains protein 1 | - | Yi and Oh 2015 |
| *APBB1IP* | Amyloid beta A4 precursor protein-binding family B member 1-interacting protein | ND | ND |
| *ASAP1* | Arf-GAP with SH3 domain, ANK repeat and PH domain-containing protein 1 | ND | ND |
| *LRRFIP1* | Leucine-rich repeat flightless-interacting protein 1 | ND | ND |
| *SCRN1* | Secernin-1 | ND | ND |
| *TGM2* | Protein-glutamine gamma-glutamyltransferase | - | Yi and Oh 2015 |
| *LACTB* | Serine beta-lactamase-like protein LACTB, mitochondrial | ND | ND |
| *TGFBI* | Transforming growth factor-beta-induced protein ig-h3 | - | Bauer et al. 2011 |
| *MAP4K5* | Mitogen-activated protein kinase kinase kinase kinase 5; Mitogen-activated protein kinase kinase kinase kinase | + | Shen et al. 2006 |
| *PODXL* | Podocalyxin | ND | ND |
| *NMES1;C15orf48* | Normal mucosa of esophagus-specific gene 1 protein | ND | ND |
| *CD74* | HLA class II histocompatibility antigen gamma chain | ND | ND |
| *SUN2* | SUN domain-containing protein 2 | ND | ND |
| *RBKS* | Ribokinase | ND | ND |
| *LEPREL1* | Prolyl 3-hydroxylase 2 | ND | ND |
| *ADAMTS1* | A disintegrin and metalloproteinase with thrombospondin motifs 1 | ND | ND |
| *OCIAD2* | OCIA domain-containing protein 2 | ND | ND |
| *DHFR* | Dihydrofolate reductase | ND | ND |
| *TBC1D2* | TBC1 domain family member 2A | ND | ND |
| *UBA6* | Ubiquitin-like modifier-activating enzyme 6 | ND | ND |
| *NMI* | N-myc-interactor | ND | ND |
| *EEA1* | Early endosome antigen 1 | ND | ND |
| *S100A2* | Protein S100-A2 | ND | ND |
| *ITGAV* | Integrin alpha-V; Integrin alpha-V heavy chain; Integrin alpha-V light chain | - | Satoh et al. 2013 |
| *ERAP1* | Endoplasmic reticulum aminopeptidase 1 | ND | ND |
| *HMGCS1* | Hydroxymethylglutaryl-CoA synthase, cytoplasmic | ND | ND |
| *NDUFA3* | NADH dehydrogenase [ubiquinone] 1 alpha subcomplex subunit 3 | ND | ND |
| *MVP* | Major vault protein | ND | ND |
| *NANS* | Sialic acid synthase | ND | ND |
| *PARP4* | Poly [ADP-ribose] polymerase 4 | ND | ND |
| *LASP1* | LIM and SH3 domain protein 1 | ND | ND |
| *EPHA2* | Ephrin type-A receptor 2 | + | Reddy et al. 2007 |
| *ATP2C1* | Calcium-transporting ATPase type 2C member 1; Calcium-transporting ATPase | ND | ND |
| *DPP9* | Dipeptidyl peptidase 9 | + | Shen et al. 2006 |
| *IMMT* | MICOS complex subunit MIC60 | ND | ND |
| *NF2* | Merlin | ND | ND |
| *STXBP2* | Syntaxin-binding protein 2 | ND | ND |
| *NCEH1* | Neutral cholesterol ester hydrolase 1 | ND | ND |
| *S100A11* | Protein S100-A11;Protein S100-A11, N-terminally processed | ND | ND |
| *ENAH* | Protein enabled homolog | ND | ND |
| *YWHAH* | 14-3-3 protein eta | ND | ND |
| *RIPK1* | Receptor-interacting serine/threonine-protein kinase 1 | - | Olagnier et al. 2014 |
| *GLUD1;GLUD2* | Glutamate dehydrogenase 1, mitochondrial; Glutamate dehydrogenase 2, mitochondrial | ND | ND |
| *AP2B1* | AP-2 complex subunit beta | ND | ND |
| *DFNA5* | Non-syndromic hearing impairment protein 5 | ND | ND |
| *STAT3* | Signal transducer and activator of transcription 3; Signal transducer and activator of transcription | ND | ND |
| *PLS3* | Plastin-3 | ND | ND |
| *AKR1B1* | Aldose reductase | + | Jung et al. 2013 |
| *AP3B1* | AP-3 complex subunit beta-1 | ND | ND |

Bauer AK, Cho HY, Miller-Degraff L, Walker C, Helms K, Fostel J, Yamamoto M & Kleeberger SR. 2011 Targeted Deletion of Nrf2 Reduces Urethane-Induced Lung Tumor Development in Mice *PLoS One* **6** e26590 (doi: 10.1371/journal.pone.0026590)

Chorley BN, Campbell MR, Wang X, Karaca M, Sambandan D, Bangura F, Xue P, Pi J, Kleeberger SR, & Bell DA. 2012 Identification of novel NRF2-regulated genes by ChIP-Seq: influence on retinoid X receptor alpha *Nucleic Acids Research* **40** 7416-7429 (doi: 10.1093/nar/gks409)

Dinkova-Kostova AT & Abramov AY. 2015 The emerging role of Nrf2 in mitochondrial function*. Free Radical Biology & Medicine* **88** 179-188 (doi: 10.1016/j.freeradbiomed.2015.04.036)

Jung KA, Choi BH, Nam CW, Song M, Kim ST, Lee JY & Kwak MK. 2013 Identification of aldo-keto reductases as NRF2-target marker genes in human cells. *Toxicoogyl Letters* **218** 39-49 (doi: 10.1016/j.toxlet.2012.12.026)

Lacher SE & Slattery M. 2016 Gene regulatory effects of disease-associated variation in the NRF2 network *Current Opinion in Toxicology* **1** 71–79

Lastres-Becker I, Innamorato NG, Jaworski T, Rábano A, Kügler S, Van Leuven F & Cuadrado A. 2014 Fractalkine activates NRF2/NFE2L2 and heme oxygenase 1 to restrain tauopathy-induced microgliosis *Brain* **137** 78-91 (doi: 10.1093/brain/awt323)

# MacLeod AK, McMahon M, Plummer SM, Higgins LG, Penning TM, Igarashi K & Hayes JD. 2009 Characterization of the cancer chemopreventive NRF2-dependent gene battery in human keratinocytes: demonstration that the KEAP1-NRF2 pathway, and not the BACH1-NRF2 pathway, controls cytoprotection against electrophiles as well as redox-cycling compounds. *Carcinogenesis* 30 1571-1580 (doi: 10.1093/carcin/bgp176)

Olagnier D, Peri S, Steel C, van Montfoort N, Chiang C, Beljanski V, Slifker M, He Z, Nichols CN, Lin R et al. 2014 Cellular oxidative stress response controls the antiviral and apoptotic programs in dengue virus-infected dendritic cells. *PLoS Pathogens* **10** e1004566 (doi: 10.1371/journal.ppat.1004566)

Ooi A, Wong JC, Petillo D, Roossien D, Perrier-Trudova, V, Whitten D, Min BW, Tan MH, Zhang Z, Yang XJ et al. 2011 An antioxidant response phenotype shared between hereditary and sporadic type 2 papillary renal cell carcinoma. *Cancer Cell.* **20** 511-523 (doi: 10.1016/j.ccr.2011.08.024)

Osburn WO, Yates MS, Dolan PD, Chen S, Liby KT, Sporn MB, Taguchi K, Yamamoto M & Kensler TW. 2008 Genetic or pharmacologic amplification of nrf2 signaling inhibits acute inflammatory liver injury in mice. *Toxicological Science* **104** 218-227 (doi: 10.1093/toxsci/kfn079)

Reddy NM, Kleeberger SR, Yamamoto M, Kensler TW, Scollick C, Biswal S & Reddy SP. 2007 Genetic dissection of the Nrf2-dependent redox signaling-regulated transcriptional programs of cell proliferation and cytoprotection. *Physiological Genomics* **32** 74-81

Satoh H, Moriguchi T, Takai J, Ebina M & Yamamoto M. 2013 Nrf2 prevents initiation but accelerates progression through the Kras signaling pathway during lung carcinogenesis. *Cancer Research* **73** 4158-4168 (doi: 10.1158/0008-5472.CAN-12-4499)

Shelton LM, Lister A, Walsh J, Jenkins RE, Wong MH, Rowe C, Ricci E, Ressel L, Fang Y, Demougin P et al. 2015 Integrated transcriptomic and proteomic analyses uncover regulatory roles of Nrf2 in the kidney. *Kidney International* **88** 1261-1273 (doi: 10.1038/ki.2015.286)

Shen G, Xu C, Hu R, Jain MR, Gopalkrishnan A, Nair S, Huang MT, Chan JY & Kong AN. 2006 Modulation of nuclear factor E2-related factor 2-mediated gene expression in mice liver and small intestine by cancer chemopreventive agent curcumin. *Molecular Cancer Therapeutics.* **5** 39-51

# Wu KC, Cui JY & Klaassen CD. 2012 Effect of graded Nrf2 activation on phase-I and -II drug metabolizing enzymes and transporters in mouse liver. *PLoS One* 7 e39006 (doi: 10.1371/journal.pone.0039006)

Yang H, Magilnick N, Lee C, Kalmaz D, Ou X, Chan JY & Lu SC. 2005 Nrf1 and Nrf2 regulate rat glutamate-cysteine ligase catalytic subunit transcription indirectly via NF-kappaB and AP-1. *Molecular and Cellular Biology* **25** 5933-5946

Yeager RL, Reisman SA, Aleksunes LM & Klaassen CD. 2009 Introducing the "TCDD-inducible AhR-Nrf2 gene battery". *Toxicological Sciences* **111** 238-246 (doi: 10.1093/toxsci/kfp115)

Yi YW & Oh S. 2015 Comparative analysis of NRF2-responsive gene expression in AcPC-1 pancreatic cancer cell line *Genes Genomics* **37** 97-109.
