# Supplementary material for: Restoration of type 1 iodothyronine deiodinase expression in renal cancer cells downregulates oncoproteins and affects key metabolic pathways as well as anti-oxidative system
Source: PLoS One. 2017 Dec 22;12(12):e0190179. doi: 10.1371/journal.pone.0190179 (PMC5741248; doi:10.1371/journal.pone.0190179)
Supplement: S1 File — (DOCX) [file pone.0190179.s010.docx]

**Additional information on proteins of which expression was affected by DIO1 in renal cancer cells.**

Transketolase (TKT) is one of the PPP enzymes that generates ribose-5-phosphate (R5P) and NADPH [1]. Cancer cells require R5P for nucleotide synthesis, while NADPH is used in reductive detoxification needed for protection against oxidative stress. R5P is synthesized in the oxidative phase of the PPP in which glucose-6-phosphate dehydrogenase (G6PD) oxidizes G6P, generating NADPH and subsequently R5P. In the non-oxidative PPP phase, glyceraldehyde-3-phosphate and fructose-6-phosphate are converted to R5P. Activation of TKT leads to reversal conversion of R5P to F6P and G6P with generation of NADPH, contributing to redox homeostasis [1]. In ccRCC tumors, the metabolism of sugars is rerouted towards PPP, providing both substrates for anabolic reactions as well as antioxidative protection [2].

Nicotinamide phosphoribosyltransferase (NAMPT) is a critical enzyme of the first-rate limiting step in conversion of nicotinamide to NAD+, essential for many metabolic pathways, including NADP synthesis. Decreased expression or inhibition of NAMPT attenuates glycolysis downstream of glyceraldehyde 3-phosphate dehydrogenase, increases the activity of the non-oxidative PPP branch, and results in attenuation of the TCA cycle [3]. In ccRCC cells, NAMPT inhibition attenuates their growth [4], underscoring the significance of this enzyme for cancer cell survival.

IDH2 is a mitochondrial isoform of isocitrate dehydrogenase. In hypoxic conditions, activation of IDH2 leads to reductive carboxylation of α-ketoglutarate, supporting citrate production at the expense of NADPH that donates hydrogen for α-ketoglutarate reduction. This is the major source of citrate synthesis from glutamine [5]. In ccRCC cells, citrate is further utilized for FFA synthesis that leads to lipid accumulation in cytosol and contributes to the clear cytosolic appearance of ccRCC cells [6]. Finally, ccRCC cells are highly dependent on extracellular amino acids [7]. To sustain their high amino acids demands, ccRCC cells express increased levels of amino acid transporters [8,9].

NDUFA3 subunit of mitochondrial complex I (NADH_ubiquinone oxidoreductase). Complex I is one of the elements of the respiratory chain that contributes to the H^+^ gradient across mitochondrial inner membrane [10]. It catalyzes the first step of NADH oxidation. In ccRCC the expression of mitochondrial complex I is decreased [11]. Suppression of mitochondrial complex I activity reduces NAD+/NADH ratios in breast cancer cells and enhances their metastatic potential. In contrast, enhanced complex I activity reduces metastatic growth by inducing autophagy via mTORC/Akt involving mechanism [12].

CYP4F11 belongs to the family of cytochrome P450 monooxygenases that catalyze metabolism of fatty acids [13].

ANPEP belongs to the family of zinc metalloenzymes that preferentially cleave amino acids at neutral N-terminus of the protein. The levels of aminopeptidase N are elevated in cancers and often correlate with worse prognosis for patients. ANPEP actively contributes to cancer progression by promoting angiogenesis, migration and invasion of cancer cells. Inhibition of ANPEP activity by small compounds as well as targeting antibodies inhibits tumor progression in mouse models. Hence, ANPEP is considered as an important therapeutic target for treatment of many cancers [14]. In our study, DIO1 expression reduced ANPEP levels by nearly 9-fold (Table 2).

Depletion of TBC1D2 and NMI impairs biogenesis of autolysosomes and autophagy induction, respectively [15].

**References:**

1. Pinthus JH, Whelan KF, Gallino D, Lu JP, Rothschild N. Metabolic features of clear-cell renal cell carcinoma: mechanisms and clinical implications. Can Urol Assoc J. 2011;5: 274-282. doi: 10.5489/cuaj.10196.
2. Lucarelli G, Galleggiante V, Rutigliano M, Sanguedolce F, Cagiano S, Bufo P, et al. Metabolomic profile of glycolysis and the pentose phosphate pathway identifies the central role of glucose-6-phosphate dehydrogenase in clear cell-renal cell carcinoma. Oncotarget. 2015;6: 13371-13386.
3. Tan B, Young DA, Lu ZH, Wang T, Meier TI, Shepard RL, et al. Pharmacological inhibition of nicotinamide phosphoribosyltransferase (NAMPT), an enzyme essential for NAD+ biosynthesis, in human cancer cells: metabolic basis and potential clinical implications. J Biol Chem. 2013;288: 3500-3511. doi: 10.1074/jbc.M112.394510.
4. Abu Aboud O, Chen CH, Senapedis W, Baloglu E, Argueta C, Weiss RH. Dual and Specific Inhibition of NAMPT and PAK4 By KPT-9274 Decreases Kidney Cancer Growth. Mol Cancer Ther. 2016;15: 2119-2129. doi: 10.1158/1535-7163.MCT-16-0197
5. Ward PS, Patel J, Wise DR, Abdel-Wahab O, Bennett BD, Coller HA, et al. The common feature of leukemia-associated IDH1 and IDH2 mutations is a neomorphic enzyme activity converting alpha-ketoglutarate to 2-hydroxyglutarate. Cancer Cell. 2010;17: 225-234. doi: 10.1016/j.ccr.2010.01.020.
6. Hsieh JJ, Cheng EH. The panoramic view of clear cell renal cell carcinoma metabolism: values of integrated global cancer metabolomics. Transl Androl Urol. 2016;5: 984-986. doi: 10.21037/tau.2016.11.03.
7. van der Mijn JC, Panka DJ, Geissler AK, Verheul HM, Mier JW. Novel drugs that target the metabolic reprogramming in renal cell cancer. Cancer Metab. 2016;4: 14. doi: 10.1186/s40170-016-0154-8
8. Elorza A, Soro-Arnáiz I, Meléndez-Rodríguez F, Rodríguez-Vaello V, Marsboom G, de Cárcer G, et al. HIF2α acts as an mTORC1 activator through the amino acid carrier SLC7A5. Mol Cell. 2012;48: 681-691. doi: 10.1016/j.molcel.2012.09.017.
9. Poettler M, Unseld M, Braemswig K, Haitel A, Zielinski CC, Prager GW. CD98hc (SLC3A2) drives integrin-dependent renal cancer cell behavior. Mol Cancer. 2013;12: 169. doi: 10.1186/1476-4598-12-169.
10. Yadava N, Schneider SS, Jerry DJ, Kim Ch. Impaired mitochondrial metabolism and mammary carcinogenesis. J Mammary Gland Biol Neoplasia. 2013;18: 75-87. doi: 10.1007/s10911-012-9271-3.
11. Ellinger J, Gromes A, Poss M, Brüggemann M, Schmidt D, Ellinger N, et al. Systematic expression analysis of the mitochondrial complex III subunits identifies UQCRC1 as biomarker in clear cell renal cell carcinoma. Oncotarget. 2016;7: 86490-86499. doi: 10.18632/oncotarget.13275.
12. Santidrian AF, Matsuno-Yagi A, Ritland M, Seo BB, LeBoeuf SE, Gay LJ, et al. Mitochondrial complex I activity and NAD+/NADH balance regulate breast cancer progression. J Clin Invest. 2013;123: 1068-1081. doi: 10.1172/JCI64264.
13. Johnson AL, Edson KZ, Totah RA, Rettie AE. Cytochrome P450 ω-Hydroxylases in Inflammation and Cancer. Adv Pharmacol. 2015;74: 223-262. doi: 10.1016/bs.apha.2015.05.002
14. Wickström M, Larsson R, Nygren P, Gullbo J. Aminopeptidase N (CD13) as a target for cancer chemotherapy. Cancer Sci. 2011;102: 501-508. doi: 10.1111/j.1349-7006.2010.01826.x.
15. Carroll B, Mohd-Naim N, Maximiano F, Frasa MA, McCormack J, Finelli M, et al. The TBC/RabGAP Armus coordinates Rac1 and Rab7 functions during autophagy. Dev Cell. 2013;25: 15-28. doi: 10.1016/j.devcel.2013.03.005.
